# Astaxanthin Influence on Health Outcomes of Adults at Risk of Metabolic Syndrome: A Systematic Review and Meta-Analysis

**DOI:** 10.3390/nu14102050

**Published:** 2022-05-13

**Authors:** Leona Yuen-Ling Leung, Sidney Man-Ngai Chan, Hon-Lon Tam, Emily Sze-Wan Wong

**Affiliations:** 1 The Ronin Institute, Montclair, NJ 07043, USA; leonaleung@ronininstitute.org; 2 Hong Kong Food Science and Technology Association, Hong Kong, China; 3 Canadian Academy of Independent Scholars, Vancouver, BC V6B 5K3, Canada; 4 School of Science and Technology, Hong Kong Metropolitan University, Hong Kong, China; mnchan@hkmu.edu.hk; 5 Education Department, Kiang Wu Nursing College of Macau, Macau 999078, China; 6 School of Nursing, The Hong Kong Polytechnic University, Hung Hom, Hong Kong, China

**Keywords:** astaxanthin, cardiometabolic disease, metabolic syndrome, systematic review, meta-analysis

## Abstract

The use of medication is effective in managing metabolic syndrome (MetS), but side effects have led to increased attention on using nutraceuticals and supplements. Astaxanthin shows positive effects in reducing the risk of MetS, but results from individual studies are inconclusive. This systematic review summarizes the latest evidence of astaxanthin in adults with risk factors of MetS. A systematic search of English and Chinese randomized controlled trials in 14 electronic databases from inception to 30 June 2021 was performed. Two reviewers independently screened the titles and abstracts, and conducted full-text review, quality appraisal, and extraction of data. Risk of bias was assessed by PEDro. A total of 7 studies met the inclusion criteria with 321 participants. Six studies were rated to have excellent methodological quality, while the remaining one was rated at good. Results show marginal effects of astaxanthin on reduction in total cholesterol and systolic blood pressure, and a significant attenuating effect on low-density lipoprotein cholesterol. Further robust evidence is needed to examine the effects of astaxanthin in adults at risk of MetS.

## 1. Introduction

Metabolic syndrome (MetS) is also known as syndrome X or the deadly quartet. A Swedish physician establshed the concept in the 1920s. The meaning of MetS was modified and revised by various scholars and professional organizations [1,2] for over 70 years. Until 1998, there existed a unified operational definition of MetS coined by World Health Organisation (WHO) [1,2]. In subsequent years, at least seven professional bodies further revised the definition with risk factors of cardiovascular and metabolic diseases, such as hypertension, dyslipidemia, obesity, and hyperglycemia (Table 1) [3]. Individuals with at least 3 or more criteria of the above risk factors are diagnosed as MetS. The global prevalence of MetS ranges from 10% to 84%, mostly affecting developed countries [4]. For example, a recent significant health concern in Japan is that half of the males and one-fifth of females aged 40 to 74 years suffer from MetS or pre-MetS [5]. MetS increases the risk of sudden cardiac death by 70% [6], the risk of cardiovascular events by twofold, and the risk of Type 2 diabetes mellitus (T_2_DM) by fivefold [3], thus raising healthcare costs [7].

Single or combination use of medications, regular physical activities, and/or dietary management are imperative in managing risk factors of MetS [5,7,8]. However, numerous adverse effects and the high cost of medication treatment [9], failing to meet the minimal recommended level of physical exercise [8], hard-to-follow diet regime [5], and requiring strong mind control to change the mindset and control thoughts [9] were reported. Therefore, cost-effective approaches on using bioactive compounds [10], nutraceuticals and supplements on prevention and treatment of various chronic diseases such as T_2_DM emerged. In addition, there are increased concerns about the use of bioactive compounds [10], nutraceuticals, and supplements for managing MetS [11].

Astaxanthin (AST), a natural carotenoid, shows a very strong antioxidant effect that is 14, 65, and 54 times higher than that of vitamin E, C, and ß-carotene, respectively [12]. The compound is commonly found in various aquatic animals, including salmon, shrimp, and crustaceans. Moreover, the most abundance source of AST is microalgal species *Haematococcus pluvialis* [13]. Donoso et al. [14] revealed that AST has numerous beneficial effects such as protecting the cardiovascular system, maintaining healthy vision, enhancing the immune system, improving skin condition, managing diabetic problems, and protecting the nervous system. Hence, the compound is commonly used globally as a supplement, including in Japan, South Korea, Sweden, and the US with global market size of more than USD 110 million in 2018 [15]. In addition, there is an exponential increase in the number of studies related to AST on health, beauty, and safety issues. Brendler and Williamson [15] reviewed the safety issues of 87 AST clinical trials on humans, and no serious adverse effects were reported, even at very high dosage (i.e., 45 mg daily which is about 2 times of the highest daily recommended dosage). However, there is a lack of systematic review (SR) and meta-analysis on investigating the effectiveness of AST in managing the risk factors of MetS on various dosages and durations.

This SR focuses on the use of AST in adults with risk factors of MetS. The objectives were to (i) discuss the effects of physiological (primary) outcomes on the use of AST; (ii) evaluate the effects of various dosages, durations, and frequencies of AST administration; and (iii) report on the adherence rate (secondary outcome).

**Table 1:** Comparing criteria of metabolic syndrome of seven professional institutions.

Risk Factor	WHO (1998) [16]	EGIR (1999) [16]	AACE (2003) [1]	CDS (2004) [17]	IDF (2005) [16]	NCEP-ATP III (2005 Revision) [16]	JCDCG (2007) [18]
Core element	Insulin resistance (IGT, IFG, T_2_DM or other evidence of IR)	Hyperinsulinemia (plasma insulin > 75th percentile)	Insulin resistance (IGT, IFG)	None	Central obesity (WC): ≥90 cm (M), ≥80 cm (F)	None	None
Criteria	IR or diabetes, plus two of the five criteria below	Hyperinsulinemia, plus two of the four criteria below	IR, final diagnosis is left to physician discretion	Any three of the four criteria below	Obesity, plus two of the four criteria below	Any three of the five criteria below	Any four of the five criteria below
Obesity	Waist/hip ratio: >0.90 (M), >0.85 (F); or BMI >30 kg/m^2^	WC: ≥94 cm (M), ≥80 cm (F)	BMI >25 kg/m^2^ or WC: >40 inches (M), >35 inches (F)	BMI > 25 kg/m^2^	Central obesity already required	WC: >40 inches (M), >35 inches (F)	WC: ≥90 cm (M), ≥85 cm (F)
Hyper-glycemia	IR already required	IR already required	IR already required	Fasting glucose ≥ 110 mg/dL or Tx	Fasting glucose ≥ 100 mg/dL	Fasting glucose ≥ 100 mg/dL or Tx	Fasting glucose ≥ 110 mg/dL or with a history of T_2_DM
Dys-lipidemia	TG ≥150 mg/dL or HDL-C: <35 mg/dL (M), <39 mg/dL (F)	TG ≥117 mg/dL or HDL-C <39 mg/dL	TG ≥150 mg/dL or HDL-C: <40 mg/dL (M), <50 mg/dL (F)	TG ≥150 mg/dL or HDL-C: <35 mg/dL (M), <39 mg/dL (F)	TG ≥150 mg/dL or Tx	TG ≥150 mg/dL or Tx	TG ≥150 mg/dL
Dyslipidemia (second separate criteria)	-	-	-	-	HDL-C: <40 mg/dL (M), <50 mg/dL (F); or Tx	HDL-C: <40 mg/dL (M), <50 mg/dL (F); or Tx	HDL-C: <40 mg/dL
Hyper-tension	≥140/90 mmHg	≥140/90 mmHg or Tx	>130/85 mmHg	≥140/90 mmHg or Tx	>130/85 mmHg or Tx	>130/85 mmHg or Tx	≥130/85 mmHg or Tx
Other criteria	Microalbuminuria	-	Other features of IR	-	-	-	-

BMI: body mass index; F: female; HDL-C: high-density lipoprotein cholesterol; IFG: impaired fasting glucose; IGT: impaired glucose tolerance; IR: insulin resistance; M: male; Tx: treatment; T_2_DM: Type 2 diabetes mellitus; TG: triglyceride; WC: waist circumference.

## 2. Materials and Methods

This SR and meta-analysis was registered with the International Prospective Register of Systematic Review (PROSPERO) (CRD42020215881), established with reference to the Preferred Reporting Items for Systematic Review and Meta-Analysis Protocols (PRISMA-P) guideline.

### 2.1. Search Strategy

Medical subject heading (MeSH) and keywords were used to identify relevant studies: “astaxanthin (蝦紅素/虾青素)” or “metabolic syndrome (代謝綜合症/代谢综合征)” or “cardiometabolic disease (心臟代謝疾病)” or “blood pressure (血壓/血压)” or “blood sugar (血糖)” or “body mass index (身體質量指數/身体貭量指数)” or “waist circumference (腰圍)”. Fourteen electronic databases were searched for eligible studies, including eight English databases: the Cochrane Library (Cochrane Database of Systematic Reviews (CDSR), Cochrane Central Register of Controlled Trials (CENTRAL), Cochrane Methodology Register (CMR)), Cumulative Index to Nursing and Allied Health Literature (CINAHL), EMBASE, Google Scholar, MEDLINE, OvidSP, ProQuest, ScienceDirect; as well as six Chinese databases: Capital Medical University Library (Beijing, China), China National Knowledge Infrastructure (CNKI), Chinese Biomedical Literature database (CBM), Chinese Medical Current Content (CMCC), Union Search, and WangFang were searched from inception to 30 June 2021. ClinicalTrials.gov (accessed on 30 June 2021), and University Hospital Medical Information Network Clinical Trials Registry (UMIN-CTR) were searched for relevant and ongoing studies. In addition, hand searching was also performed to identify the reference list of related literatures or reviews. A sample search for PubMed is available as supporting information (Supplementary File S1).

### 2.2. Selection Criteria

Studies that fulfilled the following criteria were included in the current review: (1) Study design: randomized controlled trials (RCTs). (2) Participants: Mean age ≥ 18 years, irrespective of race and gender, and fulfilling any one of the risk factors of MetS defined by the WHO European Group for the Study of Insulin Resistance (EGIR), American Association of Clinical Endocrinology (AACE), Chinese Diabetes Society (CDS), International Diabetes Federation (IDF), National Cholesterol Education Program (NCEP), Adult Treatment Panel III (ATPIII), Chinese Joint Committee for Developing Chinese Guidelines (JCDGC) were regarded as the baseline of the study. Studies conducted on animals, children, and adolescents, and those that were still recruiting participants were excluded in this review. (3) Intervention: studies examined the use of AST in any dosage and regime, and the control group included the use of placebo that did not contain AST or did not receive any intervention. (4) Outcome measures: Studies involving at least one measure of the risk factors of MetS: (i) waist circumference (WC), body mass index (BMI), blood pressure (systolic and diastolic blood pressure) (BP), glycosylated hemoglobin level (HbA1c), fasting blood glucose (FBG), lipid profile (total cholesterol (TC), triglyceride, high-density lipoprotein cholesterol (HDL-C), low-density lipoprotein cholesterol (LDL-C)), insulin resistance level, and (ii) adherence rate were included. We also contacted the authors for clarification of some unpublished data.

### 2.3. Selection Process

All selected studies were extracted and imported to Rayyan QCRI web tool [19], and checked for duplicates. Two independent reviewers (L.L.Y.L. and H.L.T.) assessed the titles and abstracts of all potential studies identified by search strategy. Full texts were obtained if the abstract had provided adequate information regarding inclusion and exclusion criteria. Next, the full text of all retrieved studies was evaluated on the basis of participants, interventions, outcomes measures, and type of study. Decisions to include studies in the review were by the same independent reviewers. Two independent reviewers employed the self-designed eligibility verification checklist (Supplementary File S2) to conduct the selection process. Disagreements between the two reviewers were resolved by discussion. Disputes were resolved by a third reviewer (E.S.-W.W.) through discussion.

### 2.4. Data Collection Process and Data Extraction

Data extraction was performed on a pilot-tested standardized form (Supplementary File S3) modified from the JBI data extraction form for experimental or observational studies [20] on Microsoft Excel by the first and third authors, and the accuracy of the information was checked by the second and fourth authors. Two reviewers independently performed the data extraction process by employing a three-step approach to select studies that potentially met the inclusion criteria. The following information was extracted: first author’s name, study location, year of publication, sample size, participant information, intervention details, outcome measures (all time points), and authors’ conclusions.

### 2.5. Data Synthesis and Statistical Analysis

Meta-analysis was performed when at least two studies had evaluated the same outcome. All quantitative data from selected studies were pooled in statistical meta-analysis by using RevMan 5.4. All results were subjected to double data entry. Mean difference (MD) and its corresponding 95% confidence interval (CI) for each study were used to estimate the pooled effects of the included studies on each continuous variable measured on the same instrument. Unit conversions performed on those outcome measures are presented in different units (e.g., mmol/L to mg/dL). Heterogeneity was statistically assessed by using I^2^, taking >75%, 50%, and <25% for high, moderate, and low heterogeneity, respectively [21,22]. The random effect was applied to count effect sizes to provide more balance on individual study weight; hence, the summary effect was more conservative [23,24].

### 2.6. Risk of Bias in Individual Studies

Two reviewers (L.L.-Y.L. and H.-L.T.) independently assessed the risk of bias (RoB) of the included studies. All studies were appraised by using the Physiotherapy Evidence Database (PEDro) [25]. It included a total of 11 items, and each satisfied item contributed 1 point to the total score except the first item. Only items 2 to 10 were rated, and the total score ranged between 0 and 10 points. The included studies were rated as “poor”, “fair”, “good” or “excellent” with scores < 4, 4 to 5, 6 to 8, or 9 to 10, respectively. The items used for the assessment of the included studies were as follows: eligibility criteria and source; random allocation; concealed allocation; baseline comparability; blinding of participants, therapists and assessors; adequate follow-up; intention-to-treat analysis; between-group statistical comparisons; and reporting of point measures of variability (Supplementary File S4). Disagreements between the two reviewers were resolved by discussion and recommendations from the third reviewer (E.S.-W.W.).

## 3. Results

### 3.1. Study Selection

**Figure 1:**
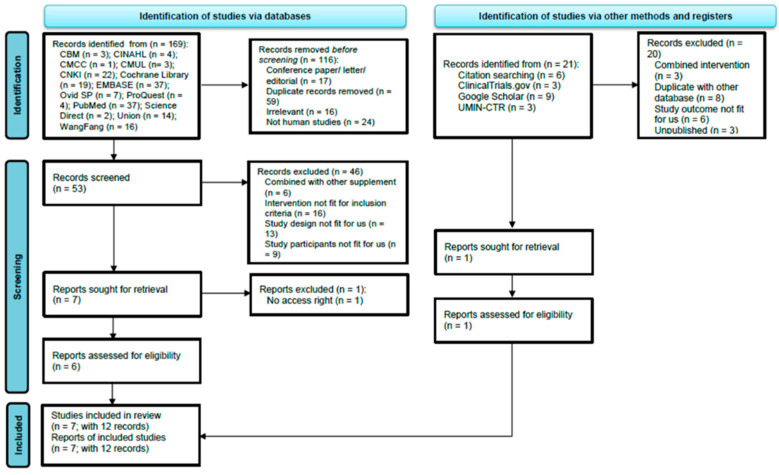
PRISMA flow diagram of searching and selection of the articles. Note. CBM: Chinese Biomedical Literature Database; CINAHL = Cumulative Index of Nursing and Allied Health Literature; CMCC = Chinese Medical Current Content; CMUL = Capital Medical University Library; CNKI: China National Knowledge; EMBASE: Excerpta Medica database; UMIN-CTR: University Hospital Medical Information Network Clinical Trials Registry.

The flow of the literature search and the selection process are summarized in Figure 1. A total of 190 records in English and Chinese were identified from electronic databases and other sources. The full-text screening identified 7 studies (317 subjects completed the trials) eligible for inclusion, and a total of 183 papers were excluded due to non-RCT studies, combined intervention, and duplicate records. The list of excluded studies is presented in Supplementary File S5. For interventional studies involving animals or humans and studies that required ethical approval, the corresponding ethical approval authorities and ethical approval code are listed.

### 3.2. Study Characteristics

The overall characteristics of the included RCTs are presented in Table 2. All studies were double-blind and published in English. The included studies were published between 2009 and 2018. Two were conducted in Japan [26,27], and the rest were conducted in Iran [28], Finland [29], Korea [30], Australia [31], and Canada [32]. The included studies comprised 17 treatment arms with a total of 321 participants (185 participants in the AST arm and 136 participants in the control arm). The sample sizes varied from 27 [30] to 63 [32]. The daily dosage of AST varied from 0.16 [29] to 20 mg [30]. Two studies reported outcomes in terms of different dosages of AST [26,27]. The range of intervention periods was from 8 weeks [28,29] to 12 months [31]. Participant characteristics included carpal tunnel syndrome (CTS), T_2_DM, obesity, mild and moderate hypertension, and having undergone renal transplantation. Reported outcomes included the risk factors of MetS: systolic blood pressure (SBP), diastolic blood pressure (DBP), body mass index (BMI), fasting blood glucose (FBG), lipid profile (e.g., total cholesterol (TC), high-density lipoprotein cholesterol (HDL-C), low-density lipoprotein cholesterol (LDL-C), triglyceride (TG)) and waist circumference (WC).

### 3.3. Quality of the Included Studies

The methodological quality of included studies was assessed with the PEDro scale as shown in Table 3. Scores of all included studies ranged from 8 to 10 with an average score of 9.29. The overall quality of the included studies was good to excellent. All included studies performed randomization, concealed allocation, blinding of participants, between-group comparison, point measure and measures of variability with similar baseline characteristics and more than 85% retention. Six out of the seven included studies [26,28,29,30,31,32] involved the blinding of the therapists, and only four [28,29,31,32] included the blinding of assessors throughout.

### 3.4. Meta-Analsysis Results—Primary Outcomes

#### 3.4.1. Body Mass Index (BMI)

**Figure 2:**
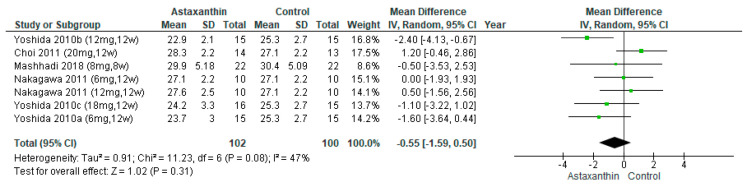
Forest plot of effect of astaxanthin on body mass index [26,27,28,30]. Bold means total data.

Four studies [26,27,28,30] evaluated the effects of AST on body mass index (BMI). These studies involved 162 subjects (AST group = 102, control group = 60). Figure 2 indicates that AST did not reduce BMI (MD = −0.55; 95% CI = −1.59, 0.50; *I*^2^ = 47%; *p* = 0.31), Supplementary File S6 Figure S2a,b indicate the subgroup analysis on different dosage and duration of AST, respectively.

#### 3.4.2. Fasting Blood Glucose (FBG)

**Figure 3:**
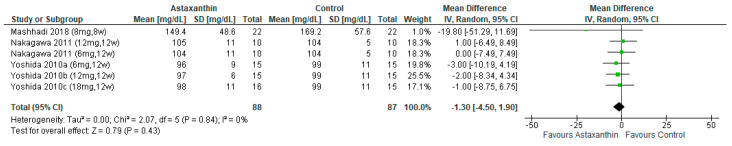
Forest plot of the effect of astaxanthin on fasting blood glucose [26,27,28]. Bold means total data.

Three studies [26,27,28] evaluated the effects of AST on FBG. The pooled result showed no significant effects of AST on FBG reduction (MD = −1.30; 95% CI = −4.50, 1.90; *I*^2^ = 0%; *p* = 0.43; Figure 3), Supplementary File S6 Figure S3a,b indicate the subgroup analysis on different dosage and duration of AST, respectively.

#### 3.4.3. Systolic Blood Pressure (SBP)

**Figure 4:**
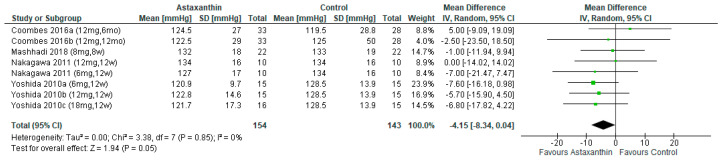
Forest plot of the effect of astaxanthin on systolic blood pressure [26,27,28,31]. Bold means total data.

Four studies [26,27,28,31] included 297 subjects (AST group = 154 subjects, control group = 143) were pooled for analysis. The result showed AST had marginally significant effect on SBP reduction (MD = −4.15; 95% CI = −8.34, 0.04; *I*^2^ = 0%; *p* = 0.05; Figure 4). Subgroup analysis exhibited that the SBP was reduced significantly when AST was administered for more than 8 weeks (MD = −4.69; 95% CI = −9.23, −0.16; *I*^2^ = 0%; *p* = 0.04) (Supplementary File S6 Figure S4a). Supplementary File S6 Figure S4a,b indicate the subgroup analysis on different dosage and duration of AST.

#### 3.4.4. Diastolic Blood Pressure (DBP)

**Figure 5:**
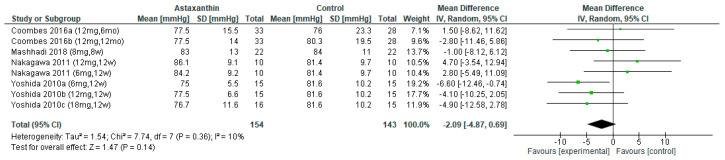
Forest plot of the effect of astaxanthin on diastolic blood pressure [26,27,28,31]. Bold means total data.

The outcome of DBP was reported in four studies [26,27,28,31], which involved 297 subjects (AST group = 154 subjects, control group = 143). However, the pooled result did not reveal any significant DBP reduction after the administration of AST (MD = −2.09; 95% CI = −4.87, 0.69; *I*^2^ = 10%; *p* = 0.14; Figure 5), Supplementary File S6 Figure S5a,b indicate the subgroup analysis on different dosage and duration of AST, respectively.

#### 3.4.5. Total Cholesterol (TC)

**Figure 6:**
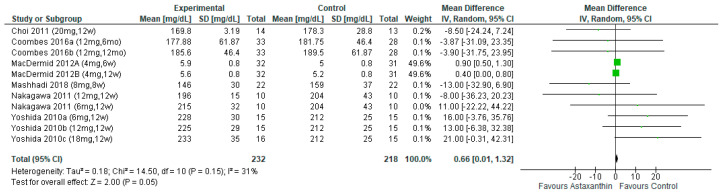
Forest plot of the effect of astaxanthin on total cholesterol [26,27,28,30,31,32]. Bold means total data.

The pooled result of seven studies [26,27,28,29,30,31,32] involving 450 subjects showed marginal significant difference between AST and the control group (MD = 0.66; 95% CI = 0.01, 1.32; *I*^2^ = 31%; *p* = 0.05; Figure 6) on TC reduction. Moreover, significant differences were found in TC for subjects consumed AST more than 8 weeks and dosages ranging from ≤6 mg/day on reducing of TC (Supplementary File S6 Figure S6a,b).

#### 3.4.6. High-density Lipoprotein Cholesterol (HDL-C)

**Figure 7:**
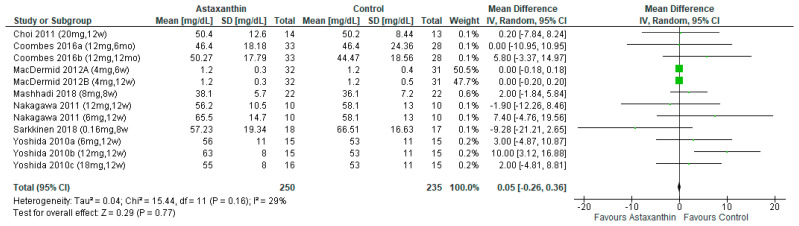
Forest plot of the effect of astaxanthin on high-density lipoprotein cholesterol [26,27,28,29,30,31,32]. Bold means total data.

No significant pooled effects on HDL-C reduction were found in seven studies [26,27,28,29,30,31,32], regardless the various subgroup analysis on different dosages and durations (MD = 0.55; 95% CI = −0.26, 0.36; *I*^2^ = 29%; *p* = 0.77; Figure 7) (Supplementary File S6 Figure S7a,b).

#### 3.4.7. Low-density Lipoprotein Cholesterol (LDL-C)

**Figure 8:**
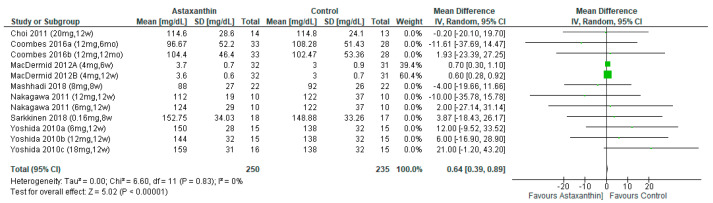
Forest plot of the effect of astaxanthin on low-density lipoprotein cholesterol [26,27,28,29,30,31,32]. Bold means total data.

The outcome of LDL-C was reported in seven studies [26,27,28,29,30,31,32], involving 485 subjects. However, AST significantly increased the level of LDL-C (MD = 0.64; 95% CI = 0.64, 0.89; *I*^2^ = 0%; *p* < 0.00001; Figure 8), regardless the duration of consumption and dosage of administration (Supplementary File S6 Figure S8a,b).

#### 3.4.8. Triglyceride (TG)

**Figure 9:**
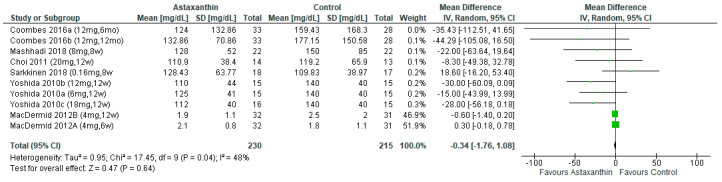
Forest plot of the effect of astaxanthin on triglyceride [26,28,29,30,31,32]. Bold means total data.

Six studies [26,28,29,30,31,32] evaluated the effects of AST on TG with a total of 445 subjects. The results showed no significant difference (MD = −0.34; 95% CI = −1.76, 1.08; *I*^2^ = 48%; *p* = 0.64; Figure 9) between the AST group (*n* = 230) and the control group (*n* = 215). However, subgroup analysis indicated significant attenuating effects of AST on TG for consumption more than 8 weeks (MD = −15.25; 95% CI = −29.75, −0.75; *I*^2^ = 46%; *p* = 0.04) and the dosage between 7 and 12 mg/day (MD = −30.08; 95% CI = −51.80, 8.36; *I*^2^ = 0%; *p* = 0.007) (Supplementary File S6 Figure S9a,b).

#### 3.4.9. Waist Circumference (WC)

Only one study [30] involving 27 participants (AST group = 14, control group = 13) reported that the use of AST could significantly reduce WC at week 12. However, the sample size of the study was very small.

### 3.5. Secondary Outcome

Adherence was the secondary outcome of this review. Only one study [30] reported this outcome. The result showed that the adherence rate at week 12 was 93.4% and 92.9% for the AST and control groups, respectively.

## 4. Discussion

In this systematic review, an extensive database search was conducted, and a validated appraisal tool was used to evaluate the effectiveness of AST in alleviating the risk factors of MetS. Results indicate that AST was effective in reducing SBP, TC, and LDL-C, where the former two had marginal statistical significant results (*p* = 0.05), and the latter showed statistical significance (*p* < 0.05). Subjects’ SBP decreased when dosed with AST for more than 8 weeks. AST induced attenuating effects on TC for using AST at the dosages of ≤6 mg/day for less than 8 weeks. Consuming AST at the dosages of ≤6 mg/day showed statistically significant effects on LDL-C for more than 8 weeks but not less than 8 weeks. In addition, AST was effective in the reduction of TG when subjects consumed dosage between 7 and 12 mg/day for more than 8 weeks.

Yanai, et al. [33] supported our findings of AST reducing SBP, as AST was associated with the enhancement of superoxide scavenging and vasorelaxation. For the lipid profile, a study conducted by Choi et al. [30] revealed that AST aided in improving the lipid profile by speeding the process of dissolution and controlling the production of LDL. On the other hand, contradictory studies to the results in this SR were also found. Xia et al. [34] reported that AST indicated improvement in HDL but not other lipid profiles, blood pressure, and serum glucose. Another related SR conducted by Ursoniu et al. [12] concluded that there was no significant effect of AST on lipid profile and serum glucose. However, these two reviews [12,34] were focused on the effects on physical biomarkers, while the present study was disease-based with a focus on MetS. In addition, there was a 12-week study [30] reported an adherence rate of over 92% in both groups; however, there was no information on the strategies on sustained adherence rate.

### 4.1. Reporting Biases

Publication bias may occur since results of some clinical trials conducted by pharmaceutical or health products companies that are registered in WHO International Clinical Trial Registry Platform, and UMIN-CTR Clinical Trial, were not published. This type of publication bias may lead to spurious beneficial treatment effects or missing some important adverse effects. To deal with this bias, we searched the gray literature and those potential studies. However, the clinical trials studying this topic are still very limited. Among the seven included studies, only one [29] mentioned the allocation concealment of subjects in the trial, while the six other studies [26,27,28,30,31,32] only briefly mentioned that the trials belong to RCTs, which may have led to randomization bias. Three studies [26,27,30] did not delineate the blinding of the outcome assessors, since the outcome assessors might alter the assessment intentionally, and measurement bias might occur [35].

### 4.2. Strengths

This is the first SR to investigate the effects of AST on risk factors of MetS with a registered SR protocol. Subgroup analyses, and changes between before and after intervention treatments had been performed to explore the effectiveness of AST with different dosages and duration. An extensive and comprehensive search strategy was adopted to identify studies in multiple databases. In addition, in this SR, study selection and data extraction were separately conducted by two independent reviewers, and a third reviewer was consulted if necessary to minimize errors and potential bias [24]. All included studies had good-to-excellent quality in terms of methodology (PEDro = 8 to 10).

### 4.3. Limitations

There were several limitations of this SR. First, variations across the included studies with different dosages and different health conditions led to moderate heterogeneity in some results. Second, the dietary patterns and activities of subjects in some individual studies were not mentioned. Moreover, there is no definitive dosage and duration of AST for adults at risk of MetS. The total number of participants was small, which could have led to wide confidence intervals and worse result precision [24]. The covered identified studies were only those in English and Chinese, which may have led to publication bias, language bias, and missing studies published in other languages. However, the search of 14 databases may have reduced this bias.

### 4.4. Implication for Future Studies

There are several implications for future studies. First, different intervals of intervention outcomes can be measured for the better identification of the effects and progress of AST, such as increasing the duration of all included studies to more than 8 weeks. Intervention outcome measurements can be extended to 1 month or longer after the completion of the intervention to assess the sustainable effect of AST. Furthermore, a more rigorous RCT with a large sample is needed to further confirm findings. In addition, dietary and medication records should be properly kept for the identification of any confounding factors affecting outcomes.

## 5. Conclusions

This SR indicated the potential effects of AST on improving SBP, TC, and LDL, although the effectiveness of AST on managing risk factors of MetS was still inconclusive because of the limited number of included studies. Rigorous large-scale RCT on human subjects should be conducted to further confirm the effectiveness of AST on adults at risk of MetS.

## Figures and Tables

**Table 2:** Review characteristics of included studies (*n* = 7).

	Design, Country, No. Study Site	Number of Participants (% Female)	Mean Age in Years (SD)	Study Population	Primary Aim	Outcomes	Attrition Rate (%)	ITT	Protocol
Choi et al. (2011) [30]	Double-blind RCT Korea 1 study site	27 (*n* = 4, 14.81%)	Placebo 30.1 ± 9.5; Ix 31.1 ± 9.4	Overweight adults (aged 20–55 years; BMI > 25.0 kg/m^2^); and overweight (BMI > 25.0 kg/m^2^)	Evaluate positive effects of AST on LPs and OS state in overweight adults	At baseline and week 12: anthropometric data, LPs, apolipidprotein A1, apolipidprotein B MDA, 15-isoprostane F2t (ISP; also known as 8-epi-PGF2α, 8-iso-PGF2α, or 8-isoprostane), SOD, TAC measured to evaluate OS at baseline and at 4, 8, and 12 weeks	Adherence rate: Ix 93.4 and placebo 92.9%	27	No information
Coombes et al. (2016) [31]	Double-blind RCT Australia 2 study sites	33 (*n* = 16, 26%)	All 49.9 ± 12.2; (Placebo 50.9 ± 13.4; Ix 49.1 ± 11.2)	Age > 18 and <85 y and having undergone renal transplantation	Assess the effect of AST on arterial stiffness, OS, and inflammation in renal transplant recipients	Primary outcomes: PWV, OS (total F2-isoprostanes), and inflammation (pentraxin-3) Secondary outcomes: Vascular function, CIMT, Aix, CBP, SERV, and additional measures of OS and inflammation	3 (4.92%)	58	No information
MacDermid et al. (2012) [32]	Double-blind RCT Canada 1 study site	63 (*n* = 18, 28.57%)	Control 49 ± 9; Ix 49 ± 7	CTS clinically diagnosed by hand surgeons and supported by electrophysiological abnormality; competent to comply with treatment and complete study evaluations; aged 18–65 years	Evaluate effectiveness of food additive AST as adjunct in management of CTS	Primary outcome: severity of symptoms of CTS (symptom severity scale) Secondary outcomes: physical impairments, disability and health status measures	0 (0%)	63	No information
Mashhadi et al. (2018) [28]	Double-blind RCT Iran 1 study site	44 (*n* = 27, 61.36%)	Placebo 54 ± 8; Ix 51 ± 9.7	Adults aged 30–60 years; definitive diagnosis of T_2_DM with no insulin therapy; no pregnancy or lactation; absence of self-reported specific diseases and malignancies, kidney failure, heart disease, thyroid, and other inflammatory diseases; not taking vitamin and antioxidant supplements during the last 6 months; and no smoking or drinking	Investigate potential effects of AST on participants with T_2_DM	Adiponectin concentration, lipid peroxidation, glycemic control, insulin sensitivity, and anthropometric indices	1 (2.38%)	43	Yes
Nakagawa et al. (2011) [27]	Double-blind RCT Japan 1 study site	30 (*n* = 15, 50%)	All 56.3 ± 5.3; (Control 56.6 ± 4.4; 6 mg/day 56.3 ± 6.6; 12 mg/day; 56.1 ± 5.1)	Healthy subjects (fifteen men and fifteen women), between 50 and 69 years of age, with a BMI of 27·5 (SD 2·1) kg/m^2^	Assess the efficacy of 12-week AST (6 or 12 mg/d) on both AST and PLOOH levels in the erythrocytes of thirty middle-aged and senior subjects	Erythrocyte AST, phospholipid hydroperoxides, blood biochemical	0 (0%)	30	No information
Sarkkinen et al. (2018) [29]	Double-blind RCT Finland 2 study sites	35 (*n* = 17, 48.57 %)	All 55.4 ± 8.6 (Placebo 55.3 ± 8.4; Ix 55.5 ± 9.0)	(1) age 18–65 years, (2) overweight female or male (BMI between 25 and 30 kg/m^2^), (3) mildly or moderately elevated BP (systolic 140–159/ diastolic 90–99 mmHg)	Compare the amount and the type of adverse events during 8-week follow-up after ingestion of krill powder preparation in comparison to ingestion of respective amount of placebo in overweight study subjects with mildly or moderately elevated BP	• Anthropometric data, BP Routine clinical chemistry and haematology (day 0 and 56) Plasma total and lipoprotein lipids; total TGs and TC with enzymatic, colorimetric test and LDL-C and HDL-C concentrations with homogenous • enzymatic colorimetric method	0 (0%)	35	Yes
Yoshida et al. (2010) [26]	Double-blind RCT Japan 1 study site	61 (*n* = 20, 32.79%)	All 44 ± 8 (18 mg/day 43.8 ± 10.4; 12 mg/day 42.8 ± 8.8; 6 mg/day 47.0 ± 7.0; 0 mg/day 44.3 ± 7.0)	Healthy subjects (41 men and 20 women) with TG levels of 120–200 mg/dL	Investigate AST consumption ameliorates dyslipidemia and the association with an increase in serum adiponectin levels	FPG, TC, TG, LDL-C, and HDL-C	0 (0%)	61	No information

Aix: augmentation index; AST: astaxanthin; BMI: body mass index; BP: blood pressure; CBP: central blood pressure; CIMT: carotid artery intima-media thickness; CTS: carpal tunnel syndrome; FPG: Fasting plasma glucose; HDL-C: high-density lipoprotein cholesterol; LDL-C: low-density lipoprotein cholesterol; LPs: lipid profiles; MDA: malondialdehyde; OS: oxidative stress; PLOOH: phospholipid hydroperoxides; PWV: aortic pulse wave velocity; RCT: randomized controlled trial; SERV: sub-endocardial viability ratio; SOD: superoxide dismutase; TAC: total antioxidant capacity; T_2_DM: Type 2 diabetes mellitus; TC: total cholesterol; TG: triglyceride.

**Table 3:** Results of PEDro Scale (*n* = 7).

Items	Choi et al. (2011) [30]	Coombes et al. (2016) [31]	MacDermid et al. (2012) [32]	Mashhadi et al. (2018) [28]	Nakagawa et al. (2011) [27]	Sarkkinen et al. (2018) [29]	Yoshida et al. (2010) [26]
1. Eligibility criteria were specified	Y	Y	Y	Y	Y	Y	Y
2. Subjects were randomly allocated to groups (in a crossover study, subjects were randomly allocated an order in which treatments were received)	Y	Y	Y	Y	Y	Y	Y
3. Allocation was concealed	Y	Y	Y	Y	Y	Y	Y
4. The groups were similar at baseline regarding the most important prognostic indicators	N	Y	Y	Y	Y	Y	Y
5. There was blinding of all subjects	Y	Y	Y	Y	Y	Y	Y
6. There was blinding of all therapists who administered the therapy	Y	Y	Y	Y	N	Y	Y
7. There was blinding of all assessors who measured at least one key outcome	N	Y	Y	Y	N	Y	N
8. Measures of at least one key outcome were obtained from more than 85% of the subjects initially allocated to groups	Y	Y	Y	Y	Y	Y	Y
9. All subjects for whom outcome measures were available received the treatment or control condition as allocated or, where this was not the case, data for at least one key outcome was analysed by “intention to treat”	Y	Y	Y	Y	Y	Y	Y
10. The results of between-group statistical comparisons are reported for at least one key outcome	Y	Y	Y	Y	Y	Y	Y
11. The study provides both point measures and measures of variability for at least one key outcome	Y	Y	Y	Y	Y	Y	Y
**Overall score**	8	10	10	10	8	10	9
**Quality**	**Good**	**Excellent**	**Excellent**	**Excellent**	**Good**	**Excellent**	**Excellent**

N: not fulfilling the criteria; Y: fulfilling the criteria; overall score (only items 2–11 were counted) < 4: poor; 4–5: fair; 6–8: good; and 9–10: excellent [25].

## Data Availability

Not applicable.

## Supplementary Materials

The following supporting information can be downloaded at: https://www.mdpi.com/article/10.3390/nu14102050/s1, File S1: Sample search strategy for PubMed; File S2: Study eligibility verification form; File S3: Data extraction sheet for systematic review; File S4: PEDro appraisal tool; File S5: List of excluded SRs; File S6: Meta-analysis results.
