# Supplementary material for: Astaxanthin Influence on Health Outcomes of Adults at Risk of Metabolic Syndrome: A Systematic Review and Meta-Analysis
Source: Nutrients. 2022 May 13;14(10):2050. doi: 10.3390/nu14102050 (PMC9148008; doi:10.3390/nu14102050)
Supplement: Supplementary file 1 [file nutrients-14-02050-s001.zip › nutrients-1719386-supplementary.pdf]

Supplementary Content

Supplementary File S1. Sample search strategy for PubMed.

Supplementary File S2. Study Eligibility Verification Form.

Supplementary File S3. Data Extraction Sheet for Systematic Review.

Supplementary File S4. PEDro Appraisal tool.

Supplementary File S5. List of excluded SRs.

Supplementary File S6. Meta-analysis results.

Supplementary File S1: Sample search strategy for PubMed.

Search	Query
#1	(astaxanthin[MeSH Subheading] OR carotenoid[MeSH Subheading] OR haematococcus[MeSH Subheading] OR xanthophyll[MeSH Subheading])
#2	(astaxanthin[Title/Abstract] OR carotenoid[Title/Abstract] OR haematococcus[Title/Abstract] OR xanthophyll[Title/Abstract])
#3	#1 OR #2
#4	Metabolic Syndrome X[MeSH Subheading]
#5	Metabolic Syndrome X[Title/Abstract] OR cardiovascular syndromes metabolic[Title/Abstract] OR x syndrome metabolic[Title/Abstract] OR cardiovascular syndrome metabolic[Title/Abstract] OR syndrome x raven[Title/Abstract] OR syndrome metabolic x[Title/Abstract] OR metabolic x syndrome[Title/Abstract] OR syndrome x metabolic[Title/Abstract] OR raven syndrome x[Title/Abstract] OR syndrome metabolic cardiovascular[Title/Abstract] OR metabolic cardiovascular syndrome[Title/Abstract] OR insulin resistance syndrome x[Title/Abstract] OR syndrome x dysmetabolic[Title/Abstract] OR syndrome x insulin resistance[Title/Abstract] OR dysmetabolic syndrome x[Title/Abstract]
#6	weight circumference[MeSH Subheading]
#7	weight circumference[Title/Abstract]
#8	body mass index[MeSH Subheading]
#9	body mass index[Title/Abstract] OR quetelet's index[Title/Abstract] OR index body mass[Title/Abstract] OR quetelet* index[Title/Abstract] OR index quetelet[Title/Abstract] OR bmi[Title/Abstract]
#10	blood pressure[MeSH Subheading]
#11	blood pressure[Title/Abstract] OR diastolic pressure[Title/Abstract] OR pressure* pulse[Title/Abstract] OR pressure* systolic[Title/Abstract] OR pressure* diastolic[Title/Abstract] OR pulse pressure*[Title/Abstract] OR systolic pressure[Title/Abstract]
#12	glycosylated hemoglobin[MeSH Subheading]
#13	glycosylated hemoglobin[Title/Abstract] OR hba1*[Title/Abstract]
#14	blood sugar[MeSH Subheading]
#15	blood sugar[Title/Abstract] OR sugar blood[Title/Abstract] OR blood glucose[Title/Abstract] OR glucose blood[Title/Abstract] OR insulin sensitiv*[Title/Abstract] OR insulin resistan*[Title/Abstract] OR glucose intoleran*[Title/Abstract] OR glucose toleran*[Title/Abstract])
#16	#4 OR #5 OR #6 OR #7 OR #8 OR #9 OR #10 OR #11 OR #12 OR #13 OR #14 OR #15
#17	#3 AND #16
#18	randomize*[Title/Abstract]
#19	#17 AND #18

Supplementary File S2: Study Eligibility Verification Form

Astaxanthin for community-dwelling adults with the risk factors of metabolic syndrome: A Systematic Review

Record No. ___________ Reviewer ____________________________ Date _____________
Author(s) / Year ____
Article Title ____
Journal / Volume (issue) / Page number ____

Inclusion Criteria of the Study

Please ✓ when appropriate

The study must have "Yes" in Category 1(a), 1(b) and 2, and must have at least one "Yes" in Category 3 and 4.

	Criteria	Yes	No
1. Type of Participants			
(a)	Adults over or equal to the age of 18 years old		
(b)	Participants are living in the community (not hospitalized or institutionalized)		
2. Type of Interventions			
	Astaxanthin as an intervention, but not used food enriched with astaxanthin or non-standardized preparations containing astaxanthin		
3. Type of Outcomes			
(a)	*Indicators of metabolic syndrome such as* Waist circumference and/or body mass index or blood pressure or glycosylated haemoglobin level or lipid profile or insulin resistance level		
(b)	Compliance to administer of astaxanthin		
4. Types of Studies			
(a)	Randomized controlled trials (RCTs)		
(b)	Non-randomized controlled trials or quasi-experimental studies		
(c)	Prospective and retrospective cohort studies		
(d)	Case-control studies		
(e)	Cross-sectional studies		

Overall appraisal: Include ❑ Exclude ❑ Seek further information ❑

Comments (Including reason for exclusion)

____

____

Supplementary File S3. Data Extraction Sheet for Systematic Review

Reviewer ______________________ Date ______________________

Author ______________________ Year ______________________

Journal ______________________ Record Number ______________________

Study Method

____

Participants

Setting ______________________

Population ______________________

Sample size and Interventions

Intervention	Sample size	Interventions
1		
2		

Clinical outcome measures

**Outcome Description**	
**Scale/measure**	

Study results

Dichotomous data

**Outcome**		
**Intervention ( )**<br>**Number / total number**		
**Intervention ( )**<br>**Number / total number**		

Continuous data

**Outcome**		
**Intervention ( )**<br>**Mean & SD (number)**		
**Intervention ( )**<br>**Mean & SD (number)**		

Authors Conclusion

____

____

Comments

____

____

<u>Supplementary File S4: PEDro Appraisal tool</u>

Items	Content	Options
**1**	Eligibility criteria were specified	Yes, No
**2**	Subjects were randomly allocated to groups (in a crossover study, subjects were randomly allocated an order in which treatments were received)	Yes, No
**3**	Allocation was concealed	Yes, No
**4**	The groups were similar at baseline regarding the most important prognostic indicators	Yes, No
**5**	There was blinding of all subjects	Yes, No
**6**	There was blinding of all therapists who administered the therapy	Yes, No
**7**	There was blinding of all assessors who measured at least one key outcome	Yes, No
**8**	Measures of at least one key outcome were obtained from more than 85% of the subjects initially allocated to groups	Yes, No
**9**	All subjects for whom outcome measures were available received the treatment or control condition as allocated or, where this was not the case, data for at least one key outcome was analysed by "intention to treat"	Yes, No
**10**	The results of between-group statistical comparisons are reported for at least one key outcome	Yes, No
**11**	The study provides both point measures and measures of variability for at least one key outcome	Yes, No

Verhagen, A. P., de Vet H. C., de Bie, R. A., Kessels, A. G., Boers, M., Bouter, L. M., & Knipschild, P. G. (1998). The Delphi list: a criteria list for quality assessment of randomised clinical trials for conducting systematic reviews developed by Delphi consensus. *Journal of Clinical Epidemiology, 51*(12),1235-1241.

Notes on administration of the PEDro scale:

All criteria **Points are only awarded when a criterion is clearly satisfied**. If on a literal reading of the trial report it is possible that a criterion was not satisfied, a point should not be awarded for that criterion.

Criteria	Description
**1**	This criterion is satisfied if the report describes the source of subjects and a list of criteria used to determine who was eligible to participate in the study
**2**	A study is considered to have used random allocation if the report states that allocation was random. The precise method of randomisation need not be specified. Procedures such as coin-tossing and dice-rolling should be considered random. Quasi-randomisation allocation procedures such as allocation by hospital record number or birth date, or alternation, do not satisfy this criterion.
**3**	*Concealed allocation* means that the person who determined if a subject was eligible for inclusion in the trial was unaware, when this decision was made, of which group the subject would be allocated to. A point is awarded for this criterion, even if it is not stated that allocation was concealed, when the report states that allocation was by sealed opaque envelopes or that allocation involved contacting the holder of the allocation schedule who was "off-site".
**4**	At a minimum, in studies of therapeutic interventions, the report must describe at least one measure of the severity of the condition being treated and at least one (different) key outcome measure at baseline. The rater must be satisfied that the groups' outcomes would not be expected to differ, on the basis of baseline differences in prognostic variables alone, by a clinically significant amount. This criterion is satisfied even if only baseline data of study completers are presented.
**4, 7-11**	*Key outcomes* are those outcomes which provide the primary measure of the effectiveness (or lack of effectiveness) of the therapy. In most studies, more than one variable is used as an outcome measure.
**5-7**	*Blinding* means the person in question (subject, therapist or assessor) did not know which group the subject had been allocated to. In addition, subjects and therapists are only considered to be "blind" if it could be expected that they would have been unable to distinguish between the treatments applied to different groups. In trials in which key outcomes are self-reported (eg, visual analogue scale, pain diary), the assessor is considered to be blind if the subject was blind.
**8**	This criterion is only satisfied if the report explicitly states *both* the number of subjects initially allocated to groups *and* the number of subjects from whom key outcome measures were obtained. In trials in which outcomes are measured at several points in time, a key outcome must have been measured in more than 85% of subjects at one of those points in time.
**9**	An *intention to treat* analysis means that, where subjects did not receive treatment (or the control condition) as allocated, and where measures of outcomes were available, the analysis was performed as if subjects received the treatment (or control condition) they were allocated to. This criterion is satisfied, even if there is no mention of analysis by intention to treat, if the report explicitly states that all subjects received treatment or control conditions as allocated.
**10**	*A between-group* statistical comparison involves statistical comparison of one group with another. Depending on the design of the study, this may involve comparison of two or more treatments, or comparison of treatment with a control condition. The analysis may be a simple comparison of outcomes measured after the treatment was administered, or a comparison of the change in one group with the change in another (when a factorial analysis of variance has been used to analyse the data, the latter is often reported as a group · time interaction). The comparison may be in the form hypothesis testing (which provides a "p" value, describing the probability that the groups differed only by chance) or in the form of an estimate (for example, the mean or median difference, or a difference in proportions, or number needed to treat, or a relative risk or hazard ratio) and its confidence interval.
**11**	A *point measure* is a measure of the size of the treatment effect. The treatment effect may be described as a difference in group outcomes, or as the outcome in (each of) all groups. *Measures of variability* include standard deviations, standard errors, confidence intervals, interquartile ranges (or other quantile ranges), and ranges. Point measures and/or measures of variability may be provided graphically (for example, SDs may be given as error bars in a Figure) as long as it is clear what is being graphed (for example, as long as it is clear whether error bars represent SDs or SEs). Where outcomes are categorical, this criterion is considered to have been met if the number of subjects in each category is given for each group.

Supplementary File S5: List of excluded SRs.

No.	Reference	Title	Reason for exclusion
1	Affenito, S. G., Lammi-Keefe, C. J., Vogel, S., Backstrand, J. R., Welch, G. W., & Adams, C. H. (1997).	Women with insulin-dependent diabetes mellitus (IDDM) complicated by eating disorders are at risk for exacerbated alternations in lipid metabolism. *European Journal of Clinical Nutrition, 51*(7), 462-466.	Intervention is not suitable for our study
2	Bakirci-Taylor, A. L., Reed, D. B., McCool, B., & Dawson, J. A. (2019).	mHealth improved fruit and vegetable accessibility and intake in young children. *Journal of Nutrition Education and Behavior, 51*(5), 556-566.	Intervention and population is not fit for our study
3	(2) Baralic, I., Andjelkovic, M., Djordjevic, B., Dikic, N., Radivojevic, N., Suzin-Zivkovic, V., Radojevic-Skodri, S., & Pejic, S. (2015).	Effects of astaxanthin supplementation on salivary IgA, oxidative stress, and inflammation in young soccer player. Evidence-based *Complementary and Alternative Medicine, 2015*, 783761, http://dx.doi.org/10.1155/2015/783761	Population is not fit for our study
4	Bianchini, F., Jaeckel, A., Vineis, P., Martinez-Garcia, C., Elmstahl, S., van Kappel, A. L., Boeing, H., Oshima, H., Riboli, E., & Kaaks, R. (2001).	Inverse correlation between alcohol consumption and lymphocyte levels of 8-hydroxydeoxyguanosine in humans. *Carcinogenesis, 22*(6), 885-890.	Study design is not suitable for our study
5	Birudaraju, D., Cherukuri, L., Kinninger, A., Chaganti, B. T., Shaikh, K., Haml, S., Flores, F., Roy, S. K., & Budoff, J. (2020).	A combined effect of cavacurcumin, eicospentaenoic acid (Omega-3s), astaxanthin and gamma –linoleic acid (omega-6) (CEAF) in healthy volunteers – a randomized, double-blind, placebo-controlled study. *Clinical Nutrition ESPEN,35*, 174-179. https://doi.org/10.1016/j.clnesp.2019.09.011	Combined intervention and wrong population
6	Canas, J. A., Lochrie, A., McGowan, A. G., Hossain, J., Schettino, C., & Balagopal, P. B. (2017).	Effects of mixed carotenoids on adipokines and abdominal adiposity in children: a pilot study. *The Journal of Clinical Endocrinology and Metabolism, 102*(6), 1983-1990.	Intervention and population is not fit for our study
7	Cartmel, B., Anderson, C., Irwin, M. L., Harrigan, M., Sanft, T., Li, F. Y., Gellermann, W., Ermakov, I. V., & Ferrucci, L. M. (2020).	Skin carotenoids are inversely associated with adiposity in breast cancer survivors. *Nutrition Research, 79*, 77-86.	Population is not fit for our study
8	Chai, W., Cooney, R. V., Franke, A. A., & Bostick, R. M. (2013).	Effects of calcium and vitamin D supplementation on blood pressure and serum lipids and carotenoids: a randomized, double-blind, placebo-controlled, clinical trial. *Annals of Epidemiology, 23*(9), 564-570.	Intervention and population is not fit for our study
9	Cicero, A. F. G., Rosticci, M., Morbini, M., Cagnati, M., Grandi, E., Parini, A., & Borghi, C. (2016).	Lipid-lowering and anti-inflammatory effects of omega 3 ethyl esters and krill oil: a randomized, cross-over, clinical trial. *Archives of Medical Science, 12*(3), 507-512.	Intervention is not suitable for our study
10	Choi, H. D., Youn, Y. K., & Shin, W. G. (2011).	Positive effects of astaxanthin on lipid profiles and oxidative stress in overweight subjects. *Plant Foods for Human Nutrition, 66*(4), 363-369.	Outcome is not fit for our study
11	Coyne, T., Ibiebele, T. I., Baade, P. D., Dobson, A., McClintock, C., Dunn, S., Leonard, D., & Shaw, J. (2005).	Diabetes mellitus and serum carotenoids: findings of a population-based study in Queensland, Australia. The American Journal of Clinical Nutrition, 82(3), 685-693.	Study design is not suitable for our study (cross-sectional survey)
12	De Castro-Oros, I., Sola, R., Valls, R. M., Brea, A., Mozas, P., Puzo, J., & Pocovi, M. (2016).	Genetic variants of LDLR and PCSK9 associated with variations in response to antihypercholesterolemic effects of armolipid plus with berberine. *PLoS One, 11*(3), e0150785. doi:10.1371/journal.pone.0150785	Outcome is not fit for our study
13	Djuric, Z., Bassis, C. M., Plegue, M. A., Ren, J., Chan, R., Sidahmed, E., Turgeon, D. K., Ruffin, M. T., Kato, I., & Sen, A. (2018).	Colonic mucosal bacteria are associated with inter-individual variability in serum carotenoid concentrations. *Journal of the Academy of Nutrition and Dietetics, 118*(4), 606-616.	Population is not fit for our study
14	Dreher, M. L. (2012).	Pistachio nuts: composition and potential health benefits. *Nutrition Reviews, 70*(4), 234-240.	Review article

No.	Reference	Title	Reason for exclusion
15	Fernandez-Jimenez, R., Santos-Beneit, G., Tresserra-Rimbau, A.,Bodega, P., de Miguel, M., de Cos-Gandoy, A., Rodriguez, C., Carral, V., Domenech, M., Estruch, R., Fernandez-Alvira, J. M., Lamuela-Raventos, R. M., & Fuster, V. (2019).	Rationale and design of school-based SI! Program to face obesity and promote health among Spanish adolescents: a cluster-randomized controlled trial. *American Heart Journal, 215*, 27-40.	Intervention and population is not fit for our study
16	Galletti, F., Fazio, V., Gentile, M., Schillaci, G., Pucci, G., Battista, F., Mercurio, V., Bosso, G., Bonaduce, D., Bambilla, N., Vitalini, C., D'Amato, M., & Giacovelli, G. (2019).	Efficacy of a nutraceutical combination on lipid metabolism in patients with metabolic syndrome: a multicenter, double blind, randomized, placebo controlled trial. *Lipids in Health and Disease, 18*(1), 66. https://doi.org/10.1186/s12944-019-1002-y	Combined intervention
17	Gann, P. H., Ma, J., Giovannucci, E., Willett, W., Sacks, F. M., Hennekens, C. H., Stampfer, M. J. (1999).	Lower prostate cancer risk in men with elevated plasma lycopene levels: results of a prospective analysis. *Cancer Research, 59*(6), 1225-1230.	Population is not fit for our study
18	Hajhashemy, Z., & Saneei, P. (2020).	Meta-analysis of astaxanthin supplementation on obesity, blood pressure, CRP, glycemic biomarkers, and lipid profile, Reanalysis is needed. *Pharmacological Research*, 105171.	Letter to the Editor
19	Huang, J. Y., Yeh, P. T., & Hou, Y. C. (2016).	A randomized, double-blind, placebo-controlled study of oral antioxidant supplement therapy in patients with dry eye syndrome. *Clinical Opthalmology, 10*, 813-820.	Intervention and population is not fit for our study
20	Itsiopoulos, C., Brazionis, L., Kaimakamis, M., Cameron, M., Best, J. D., O'Dea, K., Rowley, K. (2011).	Can the Mediterranean diet lower HbA1c in type 2 diabetes? Results from a randomized cross-over study. *Nutrition, metabolism, and cardiovascular diseases: NMCD, 20*(9), 740-747.	Intervention is not suitable for our study
21	Iwabayashi, M., Fujioka, N., Nomoto, K., Miyazaki, R., Takahashi, H., Hibino, S., Takahashi, Y., Nishikawa, K., Nishida, M., Yonei, Y. (2009).	Efficacy and safety of eight-week treatment with astaxanthin in individuals screened for increased oxidative stress burden. *Anti-aging Medicine, 6*(4), 15-21.	Study design is not suitable for our study
22	Iwamoto, T., Hosoda, K., Hirano, R., Kurata, H., Matsumoto, A., Miki, W., Kamiyama, M., Itakura, H., Yamamoto, S., & Kondo, K. (2000).	Inhibition of low-density lipoprotein oxidation by astaxanthin. *Journal of Atherosclerosis and Thrombosis, 7*(4), 216-222.	Population is not fit for our study
23	Javandoost, A., Afshari, A., Nikbakht-Jam, I., Khademi, M., Eslami, S., Nosrati, M., Foroutan-Tanha, M., Sahebkar, A., Tavalaie, S., Ghayour-Mobarhan, M., Ferns, G., Hadizadeh, F., Tabassi, A., & Mohajeri, A. (2017).	Effect of crocin, a carotenoid from saffron, on plasma cholesteryl ester transfer protein and lipid profile in subjects with metabolic syndrome: a double blind randomized clinical trial. *ARYA atherosclerosis, 13*(5), 245-252.	Intervention is not suitable for our study
24	Jorat, M. V., Tabrizi, R., Mirhosseini, N., Lankarai, K. B., Akbari, M., Heydari, S. T., Mottaghi, R., & Asemi, Z. (2018).	The effects of coenzyme Q10 supplementation on lipid profiles among patients with coronary artery disease: a systematic review and meta-analysis of randomized controlled trials. *Lipids in Health and Disease, 17*(1), 230. https://doi.org/10.1186/s12944-018-0876-4	Systematic review and meta-analysis
25	Kakutani, R., Hokari, S., Nishino, A., Ichihar, T., Sugimoto, K., Takaha, T., Kuriki, T., & Maoka, T. (2018).	Effect of oral paprika xanthophyll intake on abdominal fat in health overweight humans: a randomized, double-blind, placebo-controlled study. *Journal of Oleo Science, 57*(9), 1149-1162. doi: 10.5650/jos.ess18076	Intervention is not suitable for our study
26	Kuo, Y. C., Kou, H. L., & Liu, Y. S. (2016).	Physiological mechanism on chronic diseases of astaxanthin. *Journal of Sports Research, 26*(2), 33-42.	Study design is not suitable for our study

No.	Reference	Title	Reason for exclusion
		(Chinese)	
27	Landi, F., Martone, A. M., Salini, S., Zazzara, B., Calvani, R., Marzetti, E., Nesci, A., Di Giorgio, A., Giupponi, B., Santoro, L., & Santoliquido, A. (2019).	Effects of a new combination of medical food on endothelial function and lipid profile in dyslipidemic subjects: a pilot randomized trial. *BioMed Research International,* 1970878. https://doi.org/10.1155/2019/1970878	Combined intervention
28	Lobraico, J. M., Dilello, L. C., Buler, A. D., Cordisco, M. E., Petrini, J. R., & Ahmadi, R. (2015).	Effects of krill oil on endothelial function and other cardiovascular risk factors in participants with type 2 diabetes, a randomized controlled trial. *BMJ Open Diabetes Research & Care, 3*(1), doi:10.1136/bmjdrc-2015-000107	Intervention not fit for our study, author contacted but loss of contact on the correspondence email address
29	Manfrin, A., Trimarco, V., Manzi, M. V., Rozza, F., & Izzo, R. (2018).	A single blind, multicenter, randomized controlled trial to evaluate the effectiveness and cost of a novel nutraceutical (LopiGLIK) lowering cardiovascular disease risk. *ClincoEconomics and Outcomes Research, 10*, 601-609. http://dx.doi.org/10.2147/CEOR.S172838	Combined intervention
30	Mrazzi, G., Cacciotti, L., Pelliccia, F., Iaia, L., Volterrani, M., Caminiti, G., Sposato, B., Massaro, R., Grieco, F., & Rosano, G. (2011).	Long-term effects of nutraceutical (berberine, red yeast rice, policosanol) in elderly hypercholesterolemic patients. *Advances in Therapy, 28*(12), 1105-1113.	Combined intervention
31	McEligot, A. J., Rock, C. L., Flatt, S. W., Newman, V., Faerber, S., Pierce, J. P. (1999).	Plasma carotenoids are biomarkers of long-term high vegetable intake in women with breast cancer. *The Journal of Nutrition, 129*(12), 2258-2263.	Population is not fit for our study
32	(2) Pirro, M., Mannarino, M. R., Bianconi, V., Simental-Mendia, L. E., Bagaglia, F., Mannarino, E., Sahebkar, A. (2016).	The effects of a nutraceutical combination on plasma lipids and glucose: a systematic review and meta-analysis of randomized controlled trials. *Pharmacological research, 110*, 76-88. http://dx/doi.org/10.1016/j.phrs.2016.04.021	Systematic review and meta-analysis
33	Richer, S. P., Stiles, W., Graham-Hoffman, K., Levin, M., Ruskin, D., Wrobel, J., Park, D. W., & Thomas, C. (2011).	Randomized, double-blind, placebo-controlled study of zeaxanthin and visual function in patients with atrophic age-related macular degeneration: the Zeaxanthin and Visual Function Study (ZVF) FDA IND #78, 973. *Optometry, 82*(11), 667-680.	Intervention and population is not fit for our study
34	Ruscica, M., Gomaraschi, M., Mombelli, G., Macchi, C., Bosisio, R., Pazzucconi, F., Pavanello, C., Calabresi, L., Arnoldi, A., Sirtori, C. R., & Magni, P. (2014).	Nutraceutical approach to moderate cardiometabolic risk: results of a randomized, double-blind and crossover study with Armolipid Plus. *Journal of Clinical Lipidology, 8*(1), 61-68.	Combined intervention
35	Saito, M., Yoshida, K., Saito, W., Fujiya, A., Ohgami, K., Kitaichi, N., Tsukahara, H., Ishida, S., & Ohno, S. (2012).	Astaxanthin increases choroidal blood flow velocity. *Graefe's Archive for Clinical and Experimental Ophthalmology, 250*(2), 239-245.	Population is not fit for our study
36	Saquib, N., Natarajan, L., Rock, C. L., Flatt, S. W., Madlensky, L., Kealey, S., & Pierce, J. P. (2008).	The impact of a long-term reduction in dietary energy density on body weight within a randomized diet trial. *Nutrition and Cancer, 60*(1), 31-38.	Population is not fit for our study
37	Spiller, G. A., & Dewell, A. (2003).	Safety of an astaxanthin-rich haematococcus pluvialis algal extract: a randomized clinical trial. *Journal of Medicinal Food, 6*(1), 51-56.	No access right
38	Takagi, T., Hayashi, R., Nakai, Y., Okada, S., Miyashita, R., Yamada, M., Mihara, Y., Mizuzhima, K., Morita, M., Uchiyama, K., Naito, Y., & Itoh, Y. (2020).	Dietary intake of carotenoid- rich vegetables reduces visceral adiposity in obese Japanese men – a randomized, double-blind trial. *Nutrients, 12*(8), 2342. Doi:10.3390/nu12082342	Intervention is not suitable for our study

No.	Reference	Title	Reason for exclusion
39	Thies, F., Masson, L. F., Rudd, A., Vaughan, N., Tsang, C., Brittenden, J., Simpson, W. G., Duthie, S. Horgan, G. W., & Duthie, G. (2012).	Effect of a tomato-rich diet on markers of cardiovascular disease risk in moderately overweight, disease-free, middle-aged adults: a randomized controlled trial. *The American Journal of Clinical Nutrition, 95*(5), 1013-1022.	Intervention is not suitable for our study
40	Ursoniu, S., Sahebkar, A., Serban, M. C., & Banach, M. (2015).	Lipid profile and glucose changes after supplementation with astaxanthin: a systematic review and meta-analysis of randomized controlled trials. *Archives of Medical Science, 11*(2), 253-266.	Systematic review and meta-analysis
41	Ursoniu, S., Sahebkar, A., Serban, M. C., Antal, D., Mikhailidis, D. P., Cicero, A., Athyros, V., Fizzo, M., Rysz, J., & Banach, M. (2017).	Lipid-modifying effects of krill oil in humans: systematic review and meta-analysis of randomized controlled trials. *Nutrition Reviews, 75*(5), 361-373.	Systematic review and meta-analysis
42	Wang, L., Liu, S., Manson, J. E., Gaziano, J. M., Buring, J. E., & Sesso, H. D. (2006).	The consumption of lycopene and tomato-based food products is not associated with the risk of type 2 diabetes in women. *The Journal of Nutrition, 136*(3), 620-625.	Population is not fit for our study
43	Wang, F., He, Y., O. Santos, H., Sathian, B., C. Price, J., & Diao, J. (2020).	The effects of dehydroepiandrosterone (DHEA) supplementation on body composition and blood pressure: a meta-analysis of randomized controlled trials. *Steroids, 163*, 108710.	Meta-analysis
44	Whigham, L. D., Valeting, A. R., Hohnson, L. K., Zhang, Z., Atkinson, R. L., & Tanumihardjo,, S. A. (2012).	Increased vegetable and fruit consumption during weight loss effort correlates with increased weight and fat loss. *Nutrition & Diabetes, 2*, e48. Doi:10.1038/nutd.2012.22	Intervention is not suitable for our study
45	Wolak, T., & Paran, E. (2013).	Can carotenoids attenuate vascular aging? *Vascular Pharmacology, 59*(3), 63-66.	Intervention is not suitable for our study
46	Wolak, T., Sharoni, Y., Levy, J., Linnewiel-Hermoni, K., Stepensky, D., & Paran, E. (2019).	Effect of tomato nutrient complex on blood pressure: a double blind, randomized dose-response study. *Nutrient, 11*, 950. Doi: 10.3390/nu11050950	Review article
47	(2) Xia, W., Tang, N., Kord-Varkaneh, H., Low, T. Y., Tan, S. C., Wu, X., & Zhu, Y. (2020).	The effects of astaxanthin supplementation on obesity, blood pressure, CRP, glycemic biomarkers, and lipid profile: a meta-analysis of randomized controlled trials. *Pharmacological Research, 161*, 105113.	Review article

Included studies:

Choi, H.D.; Kim, J.H.; Chang, M.J.; Kyu-Youn, Y.; Shin, W.G. Effects of astaxanthin on oxidative stress in overweight and obese adults. *Phytotherapy research : PTR* **2011**, *25*, 1813-1818, doi:10.1002/ptr.3494.

Coombes, J.S.; Sharman, J.E.; Fassett, R.G. Astaxanthin has no effect on arterial stiffness, oxidative stress, or inflammation in renal transplant recipients: a randomized controlled trial (the XANTHIN trial). *Am J Clin Nutr* **2016**, *103*, 283-289, doi:10.3945/ajcn.115.115477.

MacDermid, J.C.; Vincent, J.I.; Gan, B.S.; Grewal, R. A blinded placebo-controlled randomized trial on the use of astaxanthin as an adjunct to splinting in the treatment of carpal tunnel syndrome. *Hand (N Y)* **2012**, *7*, 1-9, doi:10.1007/s11552-011-9381-1.

Mashhadi, N.S.; Zakerkish, M.; Mohammadiasl, J.; Zarei, M.; Mohammadshahi, M.; Haghighizadeh, M.H. Astaxanthin improves glucose metabolism and reduces blood pressure in patients with type 2 diabetes mellitus. *Asia Pac J Clin Nutr* **2018**, *27*, 341-346, doi:10.6133/apjcn.052017.11.

Nakagawa, K.; Kiko, T.; Miyazawa, T.; Carpentero Burdeos, G.; Kimura, F.; Satoh, A.; Miyazawa, T. Antioxidant effect of astaxanthin on phospholipid peroxidation in human erythrocytes. *Br J Nutr* **2011**, *105*, 1563-1571, doi:10.1017/S0007114510005398.

Sarkkinen, E.S.; Savolainen, M.J.; Taurio, J.; Marvola, T.; Bruheim, I. Prospective, randomized, double-blinded, placebo-controlled study on safety and tolerability of the krill powder product in overweight subjects with moderately elevated blood pressure. *Lipids Health Dis* **2018**, *17*, 287, doi:10.1186/s12944-018-0935-x.

Yoshida, H.; Yanai, H.; Ito, K.; Tomono, Y.; Koikeda, T.; Tsukahara, H.; Tada, N. Administration of natural astaxanthin increases serum HDL-cholesterol and adiponectin in subjects with mild hyperlipidemia. *Atherosclerosis* **2010**, *209*, 520-523, doi:10.1016/j.atherosclerosis.2009.10.012.

Supplementary File S6. Meta-analysis results

Body Mass Index (BMI)

Study or Subgroup	Astaxanthin Mean	Astaxanthin SD	Astaxanthin Total	Control Mean	Control SD	Control Total	Weight	Mean Difference IV, Random, 95% CI	Year
**2.1.1 8-week or less**									
Mashhadi 2018 (8mg,8w)	29.9	5.18	22	30.4	5.09	22	8.6%	-0.50 [-3.53, 2.53]	
**Subtotal (95% CI)**			**22**			**22**	**8.6%**	**-0.50 [-3.53, 2.53]**	
Heterogeneity: Not applicable									
Test for overall effect: Z = 0.32 (P = 0.75)									
**2.1.2 More than 8-week**									
Nakagawa 2011 (12mg,12w)	27.6	2.5	10	27.1	2.2	10	14.1%	0.50 [-1.56, 2.56]	
Nakagawa 2011 (6mg,12w)	27.1	2.2	10	27.1	2.2	10	15.1%	0.00 [-1.93, 1.93]	
Yoshida 2010a (6mg,12w)	23.7	3	15	25.3	2.7	15	14.2%	-1.60 [-3.64, 0.44]	
Yoshida 2010b (12mg,12w)	22.9	2.1	15	25.3	2.7	15	16.8%	-2.40 [-4.13, -0.67]	
Yoshida 2010c (18mg,12w)	24.2	3.3	16	25.3	2.7	15	13.7%	-1.10 [-3.22, 1.02]	
Choi 2011 (20mg,12w)	28.3	2.2	14	27.1	2.2	13	17.5%	1.20 [-0.46, 2.86]	
**Subtotal (95% CI)**			**80**			**78**	**91.4%**	**-0.55 [-1.72, 0.62]**	
Heterogeneity: Tau² = 1.18; Chi² = 11.23, df = 5 (P = 0.05); I² = 55%									
Test for overall effect: Z = 0.92 (P = 0.36)									
**Total (95% CI)**			**102**			**100**	**100.0%**	**-0.55 [-1.59, 0.50]**	
Heterogeneity: Tau² = 0.91; Chi² = 11.23, df = 6 (P = 0.08); I² = 47%									
Test for overall effect: Z = 1.02 (P = 0.31)									
Test for subgroup differences: Chi² = 0.00, df = 1 (P = 0.98), I² = 0%									

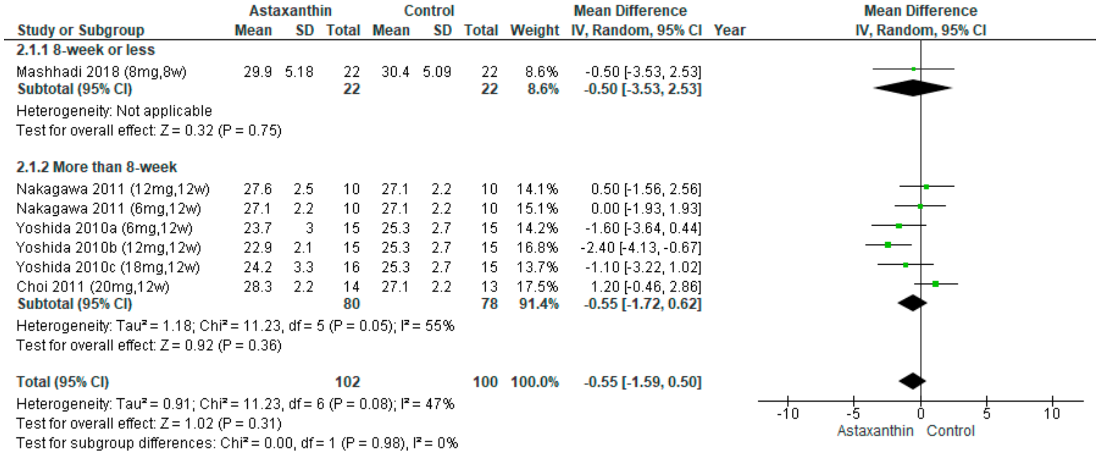

Figure S2a Subgroup meta-analysis of the effect of astaxanthin with different duration on body mass index.

Study or Subgroup	Astaxanthin Mean	Astaxanthin SD	Astaxanthin Total	Control Mean	Control SD	Control Total	Weight	Mean Difference IV, Random, 95% CI	Year
**3.1.1 less than 6mg/day**									
Nakagawa 2011 (6mg,12w)	27.1	2.2	10	27.1	2.2	10	15.1%	0.00 [-1.93, 1.93]	
Yoshida 2010a (6mg,12w)	23.7	3	15	25.3	2.7	15	14.2%	-1.60 [-3.64, 0.44]	
**Subtotal (95% CI)**			**25**			**25**	**29.4%**	**-0.76 [-2.33, 0.80]**	
Heterogeneity: Tau² = 0.25; Chi² = 1.25, df = 1 (P = 0.26); I² = 20%									
Test for overall effect: Z = 0.95 (P = 0.34)									
**3.1.2 7 - 12 mg/day**									
Nakagawa 2011 (12mg,12w)	27.6	2.5	10	27.1	2.2	10	14.1%	0.50 [-1.56, 2.56]	
Yoshida 2010b (12mg,12w)	22.9	2.1	15	25.3	2.7	15	16.8%	-2.40 [-4.13, -0.67]	
Mashhadi 2018 (8mg,8w)	29.9	5.18	22	30.4	5.09	22	8.6%	-0.50 [-3.53, 2.53]	
**Subtotal (95% CI)**			**47**			**47**	**39.5%**	**-0.91 [-2.85, 1.03]**	
Heterogeneity: Tau² = 1.65; Chi² = 4.62, df = 2 (P = 0.10); I² = 57%									
Test for overall effect: Z = 0.92 (P = 0.36)									
**3.1.3 13 mg/day or more**									
Yoshida 2010c (18mg,12w)	24.2	3.3	16	25.3	2.7	15	13.7%	-1.10 [-3.22, 1.02]	
Choi 2011 (20mg,12w)	28.3	2.2	14	27.1	2.2	13	17.5%	1.20 [-0.46, 2.86]	
**Subtotal (95% CI)**			**30**			**28**	**31.1%**	**0.15 [-2.10, 2.39]**	
Heterogeneity: Tau² = 1.70; Chi² = 2.81, df = 1 (P = 0.09); I² = 64%									
Test for overall effect: Z = 0.13 (P = 0.90)									
**Total (95% CI)**			**102**			**100**	**100.0%**	**-0.55 [-1.59, 0.50]**	
Heterogeneity: Tau² = 0.91; Chi² = 11.23, df = 6 (P = 0.08); I² = 47%									
Test for overall effect: Z = 1.02 (P = 0.31)									
Test for subgroup differences: Chi² = 0.57, df = 2 (P = 0.75), I² = 0%									

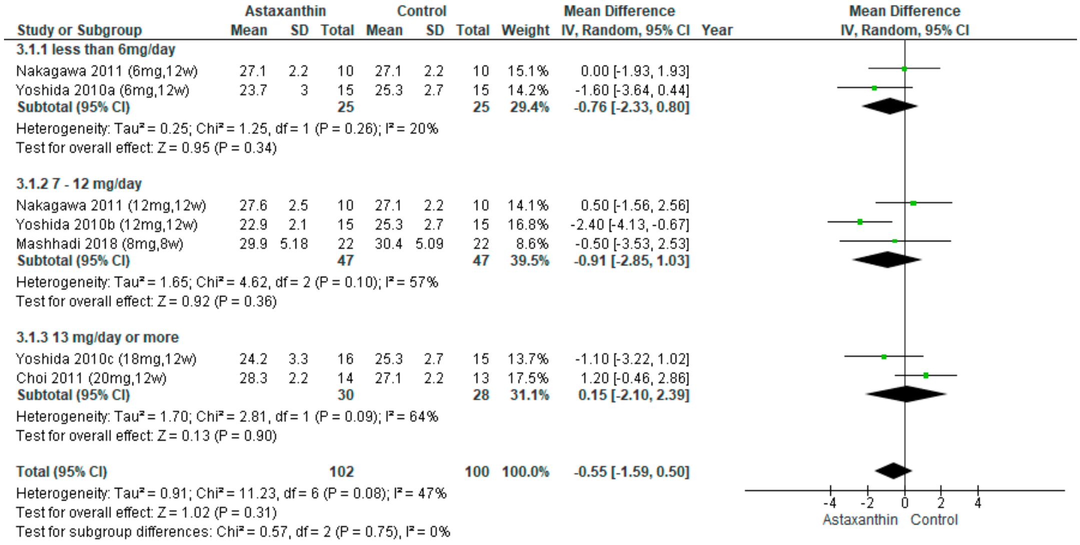

Figure S2b Subgroup meta-analysis of the effect of astaxanthin with different dosage on body mass index.

Fasting blood glucose (FBG)

Study or Subgroup	Astaxanthin Mean [mg/dL]	Astaxanthin SD [mg/dL]	Astaxanthin Total	Control Mean [mg/dL]	Control SD [mg/dL]	Control Total	Weight	Mean Difference IV, Random, 95% CI
**2.4.1 8-week or less**								
Mashhadi 2018 (8mg,8w)	149.4	48.6	22	169.2	57.6	22	1.0%	-19.80 [-51.29, 11.69]
**Subtotal (95% CI)**			**22**			**22**	**1.0%**	**-19.80 [-51.29, 11.69]**
Heterogeneity: Not applicable								
Test for overall effect: Z = 1.23 (P = 0.22)								
**2.4.2 More than 8-week**								
Nakagawa 2011 (12mg,12w)	105	11	10	104	5	10	18.3%	1.00 [-6.49, 8.49]
Nakagawa 2011 (6mg,12w)	104	11	10	104	5	10	18.3%	0.00 [-7.49, 7.49]
Yoshida 2010a (6mg,12w)	96	9	15	99	11	15	19.8%	-3.00 [-10.19, 4.19]
Yoshida 2010b (12mg,12w)	97	6	15	99	11	15	25.5%	-2.00 [-8.34, 4.34]
Yoshida 2010c (18mg,12w)	98	11	16	99	11	15	17.1%	-1.00 [-8.75, 6.75]
**Subtotal (95% CI)**			**66**			**65**	**99.0%**	**-1.10 [-4.32, 2.11]**
Heterogeneity: Tau² = 0.00; Chi² = 0.73, df = 4 (P = 0.95); I² = 0%								
Test for overall effect: Z = 0.67 (P = 0.50)								
**Total (95% CI)**			**88**			**87**	**100.0%**	**-1.30 [-4.50, 1.90]**
Heterogeneity: Tau² = 0.00; Chi² = 2.07, df = 5 (P = 0.84); I² = 0%								
Test for overall effect: Z = 0.79 (P = 0.43)								
Test for subgroup differences: Chi² = 1.34, df = 1 (P = 0.25), I² = 25.4%								

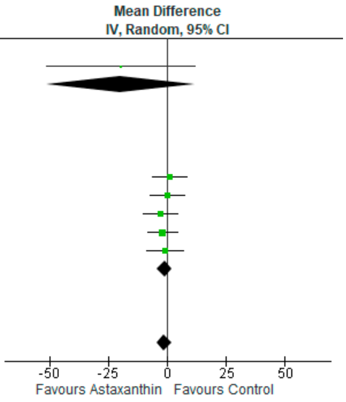

Figure S3a Subgroup meta-analysis of the effect of astaxanthin with different duration on fasting blood glucose.

Study or Subgroup	Astaxanthin Mean [mg/dL]	Astaxanthin SD [mg/dL]	Astaxanthin Total	Control Mean [mg/dL]	Control SD [mg/dL]	Control Total	Weight	Mean Difference IV, Random, 95% CI
**3.4.1 less than 6mg/day**								
Nakagawa 2011 (6mg,12w)	104	11	10	104	5	10	18.3%	0.00 [-7.49, 7.49]
Yoshida 2010a (6mg,12w)	96	9	15	99	11	15	19.8%	-3.00 [-10.19, 4.19]
**Subtotal (95% CI)**			**25**			**25**	**38.1%**	**-1.56 [-6.75, 3.63]**
Heterogeneity: Tau² = 0.00; Chi² = 0.32, df = 1 (P = 0.57); I² = 0%								
Test for overall effect: Z = 0.59 (P = 0.56)								
**3.4.2 7 - 12mg/day**								
Mashhadi 2018 (8mg,8w)	149.4	48.6	22	169.2	57.6	22	1.0%	-19.80 [-51.29, 11.69]
Nakagawa 2011 (12mg,12w)	105	11	10	104	5	10	18.3%	1.00 [-6.49, 8.49]
Yoshida 2010b (12mg,12w)	97	6	15	99	11	15	25.5%	-2.00 [-8.34, 4.34]
**Subtotal (95% CI)**			**47**			**47**	**44.8%**	**-1.19 [-5.97, 3.60]**
Heterogeneity: Tau² = 0.00; Chi² = 1.73, df = 2 (P = 0.42); I² = 0%								
Test for overall effect: Z = 0.49 (P = 0.63)								
**3.4.3 13 mg/day or more**								
Yoshida 2010c (18mg,12w)	98	11	16	99	11	15	17.1%	-1.00 [-8.75, 6.75]
**Subtotal (95% CI)**			**16**			**15**	**17.1%**	**-1.00 [-8.75, 6.75]**
Heterogeneity: Not applicable								
Test for overall effect: Z = 0.25 (P = 0.80)								
**Total (95% CI)**			**88**			**87**	**100.0%**	**-1.30 [-4.50, 1.90]**
Heterogeneity: Tau² = 0.00; Chi² = 2.07, df = 5 (P = 0.84); I² = 0%								
Test for overall effect: Z = 0.79 (P = 0.43)								
Test for subgroup differences: Chi² = 0.02, df = 2 (P = 0.99), I² = 0%								

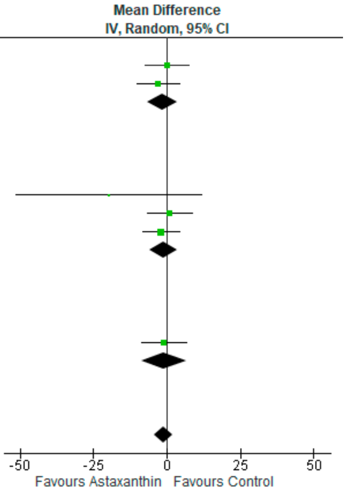

Figure S3b Subgroup meta-analysis of the effect of astaxanthin with different dosage on fasting blood glucose.

Systolic blood pressure (SBP)

Study or Subgroup	Astaxanthin Mean [mmHg]	Astaxanthin SD [mmHg]	Astaxanthin Total	Control Mean [mmHg]	Control SD [mmHg]	Control Total	Weight	Mean Difference IV, Random, 95% CI
**2.2.1 8-week or less**								
Mashhadi 2018 (8mg,8w)	132	18	22	133	19	22	14.7%	-1.00 [-11.94, 9.94]
**Subtotal (95% CI)**			**22**			**22**	**14.7%**	**-1.00 [-11.94, 9.94]**
Heterogeneity: Not applicable								
Test for overall effect: Z = 0.18 (P = 0.86)								
**2.2.2 More than 8-week**								
Coombes 2016a (12mg,6mo)	124.5	27	33	119.5	28.8	28	8.8%	5.00 [-9.09, 19.09]
Coombes 2016b (12mg,12mo)	122.5	29	33	125	50	28	4.0%	-2.50 [-23.50, 18.50]
Nakagawa 2011 (12mg,12w)	134	16	10	134	16	10	8.9%	0.00 [-14.02, 14.02]
Nakagawa 2011 (6mg,12w)	127	17	10	134	16	10	8.4%	-7.00 [-21.47, 7.47]
Yoshida 2010a (6mg,12w)	120.9	9.7	15	128.5	13.9	15	23.9%	-7.60 [-16.18, 0.98]
Yoshida 2010b (12mg,12w)	122.8	14.6	15	128.5	13.9	15	16.9%	-5.70 [-15.90, 4.50]
Yoshida 2010c (18mg,12w)	121.7	17.3	16	128.5	13.9	15	14.5%	-6.80 [-17.82, 4.22]
**Subtotal (95% CI)**			**132**			**121**	**85.3%**	**-4.69 [-9.23, -0.16]**
Heterogeneity: Tau² = 0.00; Chi² = 3.01, df = 6 (P = 0.81); I² = 0%								
Test for overall effect: Z = 2.03 (P = 0.04)								
**Total (95% CI)**			**154**			**143**	**100.0%**	**-4.15 [-8.34, 0.04]**
Heterogeneity: Tau² = 0.00; Chi² = 3.38, df = 7 (P = 0.85); I² = 0%								
Test for overall effect: Z = 1.94 (P = 0.05)								
Test for subgroup differences: Chi² = 0.37, df = 1 (P = 0.54), I² = 0%								

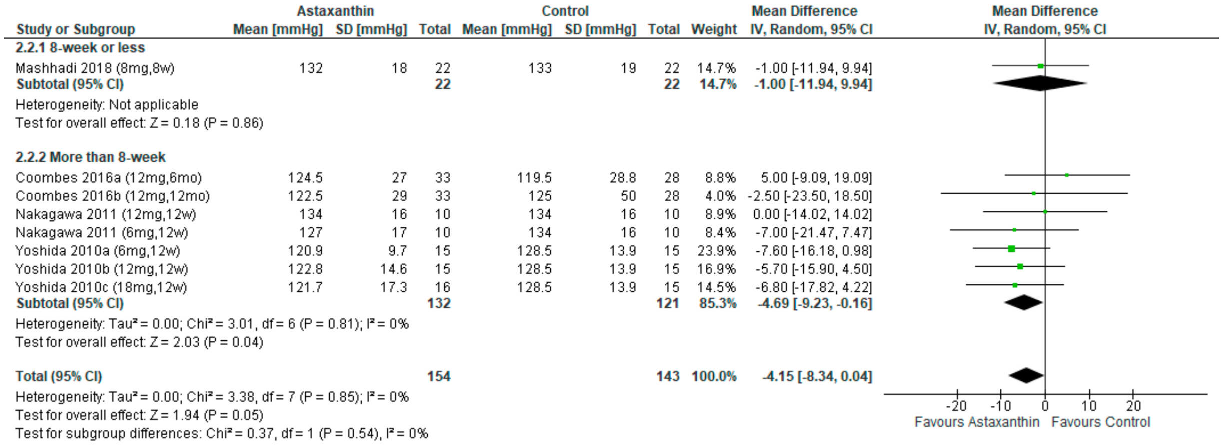

Figure S4a Subgroup meta-analysis of the effect of astaxanthin with different duration on systolic blood pressure.

Study or Subgroup	Astaxanthin Mean [mmHg]	Astaxanthin SD [mmHg]	Astaxanthin Total	Control Mean [mmHg]	Control SD [mmHg]	Control Total	Weight	Mean Difference IV, Random, 95% CI
**3.2.1 less than 6mg/day**								
Mashhadi 2018 (8mg,8w)	132	18	22	133	19	22	14.7%	-1.00 [-11.94, 9.94]
Nakagawa 2011 (6mg,12w)	127	17	10	134	16	10	8.4%	-7.00 [-21.47, 7.47]
Yoshida 2010a (6mg,12w)	120.9	9.7	15	128.5	13.9	15	23.9%	-7.60 [-16.18, 0.98]
**Subtotal (95% CI)**			**47**			**47**	**46.9%**	**-5.43 [-11.54, 0.69]**
Heterogeneity: Tau² = 0.00; Chi² = 0.92, df = 2 (P = 0.63); I² = 0%								
Test for overall effect: Z = 1.74 (P = 0.08)								
**3.2.2 7 - 12mg/day**								
Coombes 2016a (12mg,6mo)	124.5	27	33	119.5	28.8	28	8.8%	5.00 [-9.09, 19.09]
Coombes 2016b (12mg,12mo)	122.5	29	33	125	50	28	4.0%	-2.50 [-23.50, 18.50]
Nakagawa 2011 (12mg,12w)	134	16	10	134	16	10	8.9%	0.00 [-14.02, 14.02]
Yoshida 2010b (12mg,12w)	122.8	14.6	15	128.5	13.9	15	16.9%	-5.70 [-15.90, 4.50]
**Subtotal (95% CI)**			**91**			**81**	**38.6%**	**-1.60 [-8.35, 5.14]**
Heterogeneity: Tau² = 0.00; Chi² = 1.52, df = 3 (P = 0.68); I² = 0%								
Test for overall effect: Z = 0.47 (P = 0.64)								
**3.2.3 13mg/day or more**								
Yoshida 2010c (18mg,12w)	121.7	17.3	16	128.5	13.9	15	14.5%	-6.80 [-17.82, 4.22]
**Subtotal (95% CI)**			**16**			**15**	**14.5%**	**-6.80 [-17.82, 4.22]**
Heterogeneity: Not applicable								
Test for overall effect: Z = 1.21 (P = 0.23)								
**Total (95% CI)**			**154**			**143**	**100.0%**	**-4.15 [-8.34, 0.04]**
Heterogeneity: Tau² = 0.00; Chi² = 3.38, df = 7 (P = 0.85); I² = 0%								
Test for overall effect: Z = 1.94 (P = 0.05)								
Test for subgroup differences: Chi² = 0.94, df = 2 (P = 0.63), I² = 0%								

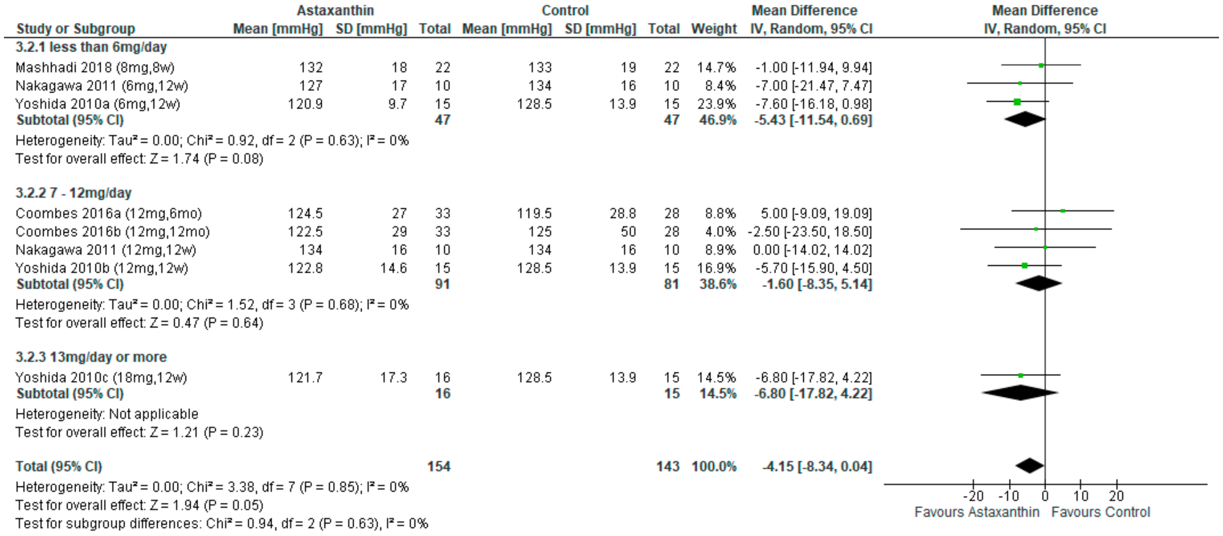

Figure S4b Subgroup meta-analysis of the effect of astaxanthin with different dosage on systolic blood pressure.

Diastolic blood pressure (DBP)

Study or Subgroup	Astaxanthin Mean [mmHg]	Astaxanthin SD [mmHg]	Astaxanthin Total	Control Mean [mmHg]	Control SD [mmHg]	Control Total	Weight	Mean Difference IV, Random, 95% CI
**2.3.1 8-weeks or less**								
Mashhadi 2018 (8mg,8w)	83	13	22	84	11	22	13.7%	-1.00 [-8.12, 6.12]
**Subtotal (95% CI)**			**22**			**22**	**13.7%**	**-1.00 [-8.12, 6.12]**
Heterogeneity: Not applicable								
Test for overall effect: Z = 0.28 (P = 0.78)								
**2.3.2 More than 8-week**								
Coombes 2016a (12mg,6mo)	77.5	15.5	33	76	23.3	28	7.1%	1.50 [-8.62, 11.62]
Coombes 2016b (12mg,12mo)	77.5	14	33	80.3	19.5	28	9.6%	-2.80 [-11.46, 5.86]
Nakagawa 2011 (12mg,12w)	86.1	9.1	10	81.4	9.7	10	10.5%	4.70 [-3.54, 12.94]
Nakagawa 2011 (6mg,12w)	84.2	9.2	10	81.4	9.7	10	10.4%	2.80 [-5.49, 11.09]
Yoshida 2010a (6mg,12w)	75	5.5	15	81.6	10.2	15	19.2%	-6.60 [-12.46, -0.74]
Yoshida 2010b (12mg,12w)	77.5	6.6	15	81.6	10.2	15	17.7%	-4.10 [-10.25, 2.05]
Yoshida 2010c (18mg,12w)	76.7	11.6	16	81.6	10.2	15	11.9%	-4.90 [-12.58, 2.78]
**Subtotal (95% CI)**			**132**			**121**	**86.3%**	**-2.13 [-5.36, 1.10]**
Heterogeneity: Tau² = 4.01; Chi² = 7.61, df = 6 (P = 0.27); I² = 21%								
Test for overall effect: Z = 1.29 (P = 0.20)								
**Total (95% CI)**			**154**			**143**	**100.0%**	**-2.09 [-4.87, 0.69]**
Heterogeneity: Tau² = 1.54; Chi² = 7.74, df = 7 (P = 0.36); I² = 10%								
Test for overall effect: Z = 1.47 (P = 0.14)								
Test for subgroup differences: Chi² = 0.08, df = 1 (P = 0.78), I² = 0%								

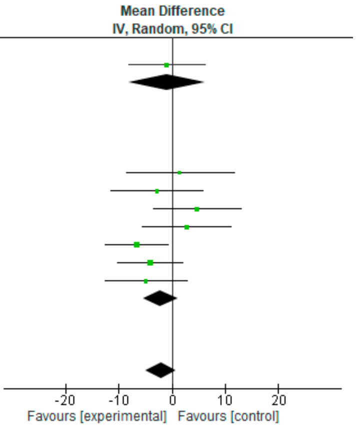

Figure S5a Subgroup meta-analysis of the effect of astaxanthin with different duration on diastolic blood pressure.

Study or Subgroup	Astaxanthin Mean [mmHg]	Astaxanthin SD [mmHg]	Astaxanthin Total	Control Mean [mmHg]	Control SD [mmHg]	Control Total	Weight	Mean Difference IV, Random, 95% CI
**3.3.1 less than 6mg/day**								
Nakagawa 2011 (6mg,12w)	84.2	9.2	10	81.4	9.7	10	10.4%	2.80 [-5.49, 11.09]
Yoshida 2010a (6mg,12w)	75	5.5	15	81.6	10.2	15	19.2%	-6.60 [-12.46, -0.74]
**Subtotal (95% CI)**			**25**			**25**	**29.6%**	**-2.37 [-11.54, 6.79]**
Heterogeneity: Tau² = 30.77; Chi² = 3.29, df = 1 (P = 0.07); I² = 70%								
Test for overall effect: Z = 0.51 (P = 0.61)								
**3.3.2 7 - 12mg/day**								
Coombes 2016a (12mg,6mo)	77.5	15.5	33	76	23.3	28	7.1%	1.50 [-8.62, 11.62]
Coombes 2016b (12mg,12mo)	77.5	14	33	80.3	19.5	28	9.6%	-2.80 [-11.46, 5.86]
Mashhadi 2018 (8mg,8w)	83	13	22	84	11	22	13.7%	-1.00 [-8.12, 6.12]
Nakagawa 2011 (12mg,12w)	86.1	9.1	10	81.4	9.7	10	10.5%	4.70 [-3.54, 12.94]
Yoshida 2010b (12mg,12w)	77.5	6.6	15	81.6	10.2	15	17.7%	-4.10 [-10.25, 2.05]
**Subtotal (95% CI)**			**113**			**103**	**58.5%**	**-0.97 [-4.42, 2.48]**
Heterogeneity: Tau² = 0.00; Chi² = 3.21, df = 4 (P = 0.52); I² = 0%								
Test for overall effect: Z = 0.55 (P = 0.58)								
**3.3.3 13mg/day or more**								
Yoshida 2010c (18mg,12w)	76.7	11.6	16	81.6	10.2	15	11.9%	-4.90 [-12.58, 2.78]
**Subtotal (95% CI)**			**16**			**15**	**11.9%**	**-4.90 [-12.58, 2.78]**
Heterogeneity: Not applicable								
Test for overall effect: Z = 1.25 (P = 0.21)								
**Total (95% CI)**			**154**			**143**	**100.0%**	**-2.09 [-4.87, 0.69]**
Heterogeneity: Tau² = 1.54; Chi² = 7.74, df = 7 (P = 0.36); I² = 10%								
Test for overall effect: Z = 1.47 (P = 0.14)								
Test for subgroup differences: Chi² = 0.86, df = 2 (P = 0.65), I² = 0%								

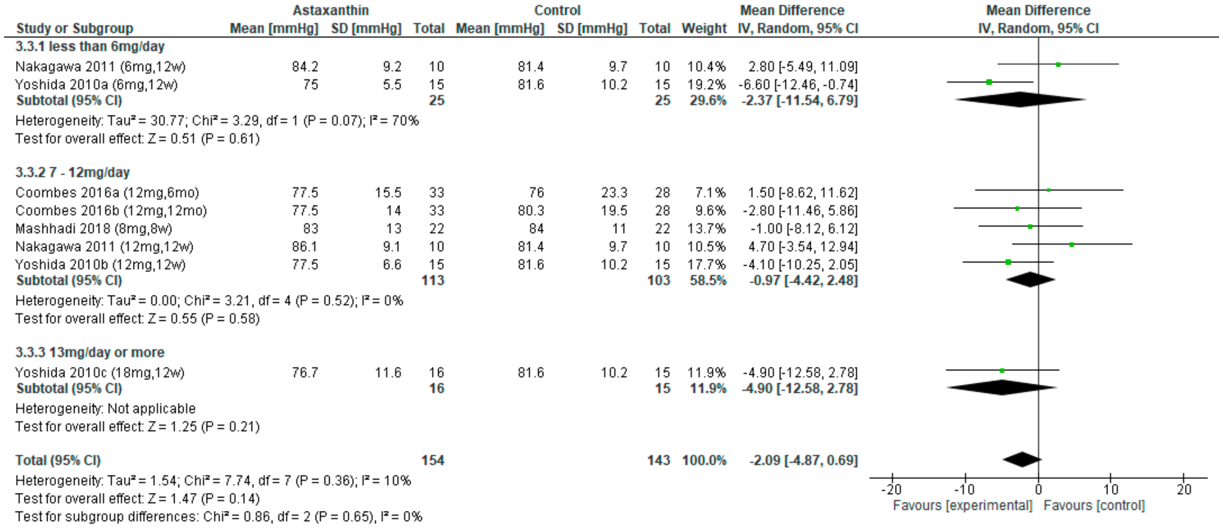

Figure S5b Subgroup meta-analysis of the effect of astaxanthin with different dosage on diastolic blood pressure.

<u>Total cholesterol (TC)</u>

Study or Subgroup	Experimental Mean [mg/dL]	Experimental SD [mg/dL]	Experimental Total	Control Mean [mg/dL]	Control SD [mg/dL]	Control Total	Weight	Mean Difference IV, Random, 95% CI
**2.5.1 8-week or less**								
MacDermid 2012A (4mg,6w)	5.9	0.8	32	5	0.8	31	49.6%	0.90 [0.50, 1.30]
MacDermid 2012B (4mg,12w)	5.6	0.8	32	5.2	0.8	31	49.6%	0.40 [0.00, 0.80]
Mashhadi 2018 (8mg,8w)	146	30	22	159	37	22	0.1%	-13.00 [-32.90, 6.90]
**Subtotal (95% CI)**			**86**			**84**	**99.3%**	**0.64 [0.09, 1.19]**
Heterogeneity: Tau² = 0.12; Chi² = 4.88, df = 2 (P = 0.09); I² = 59%								
Test for overall effect: Z = 2.28 (P = 0.02)								
**2.5.2 More than 8-week**								
Choi 2011 (20mg,12w)	169.8	3.19	14	178.3	28.8	13	0.2%	-8.50 [-24.24, 7.24]
Coombes 2016a (12mg,6mo)	177.88	61.87	33	181.75	46.4	28	0.1%	-3.87 [-31.09, 23.35]
Coombes 2016b (12mg,12mo)	185.6	46.4	33	189.5	61.87	28	0.1%	-3.90 [-31.75, 23.95]
Nakagawa 2011 (12mg,12w)	196	15	10	204	43	10	0.1%	-8.00 [-36.23, 20.23]
Nakagawa 2011 (6mg,12w)	215	32	10	204	43	10	0.0%	11.00 [-22.22, 44.22]
Yoshida 2010a (6mg,12w)	228	30	15	212	25	15	0.1%	16.00 [-3.76, 35.76]
Yoshida 2010b (12mg,12w)	225	29	15	212	25	15	0.1%	13.00 [-6.38, 32.38]
Yoshida 2010c (18mg,12w)	233	35	16	212	25	15	0.1%	21.00 [-0.31, 42.31]
**Subtotal (95% CI)**			**146**			**134**	**0.7%**	**4.94 [-3.91, 13.80]**
Heterogeneity: Tau² = 29.72; Chi² = 8.57, df = 7 (P = 0.28); I² = 18%								
Test for overall effect: Z = 1.09 (P = 0.27)								
**Total (95% CI)**			**232**			**218**	**100.0%**	**0.66 [0.01, 1.32]**
Heterogeneity: Tau² = 0.18; Chi² = 14.50, df = 10 (P = 0.15); I² = 31%								
Test for overall effect: Z = 2.00 (P = 0.05)								
Test for subgroup differences: Chi² = 0.90, df = 1 (P = 0.34), I² = 0%								

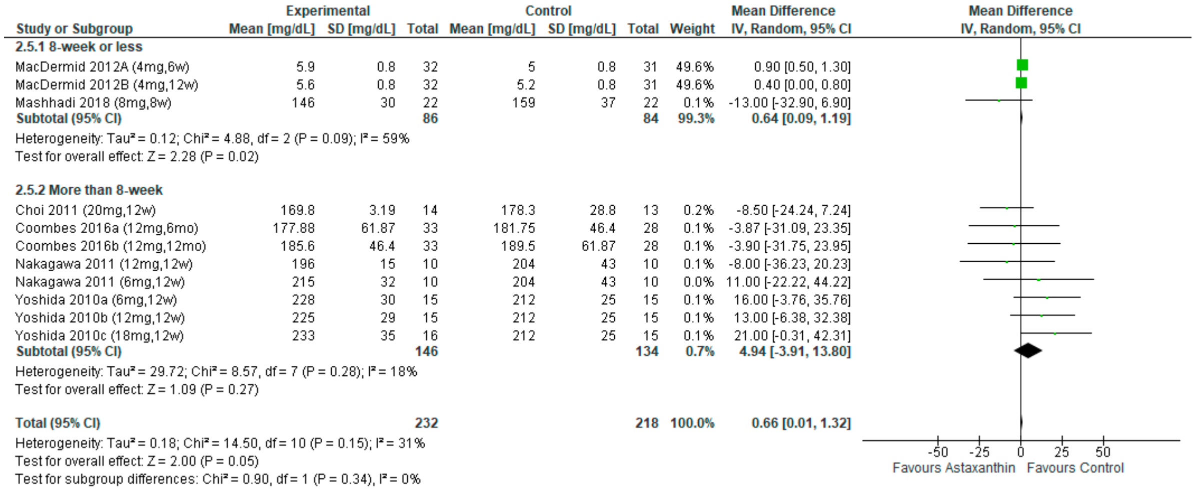

Figure S6a Subgroup meta-analysis of the effect of astaxanthin with different duration on total cholesterol.

Study or Subgroup	Experimental Mean [mg/dL]	Experimental SD [mg/dL]	Experimental Total	Control Mean [mg/dL]	Control SD [mg/dL]	Control Total	Weight	Mean Difference IV, Random, 95% CI
**3.5.1 less than 6mg/day**								
MacDermid 2012A (4mg,6w)	5.9	0.8	32	5	0.8	31	35.5%	0.90 [0.50, 1.30]
MacDermid 2012B (4mg,12w)	5.6	0.8	32	5.2	0.8	31	35.5%	0.40 [0.00, 0.80]
Nakagawa 2011 (6mg,12w)	215	32	10	204	43	10	1.3%	11.00 [-22.22, 44.22]
Yoshida 2010a (6mg,12w)	228	30	15	212	25	15	3.5%	16.00 [-3.76, 35.76]
**Subtotal (95% CI)**			**89**			**87**	**75.9%**	**0.66 [0.12, 1.21]**
Heterogeneity: Tau² = 0.11; Chi² = 5.77, df = 3 (P = 0.12); I² = 48%								
Test for overall effect: Z = 2.40 (P = 0.02)								
**3.5.2 7 - 12mg/day**								
Coombes 2016a (12mg,6mo)	177.88	61.87	33	181.75	46.4	28	1.9%	-3.87 [-31.09, 23.35]
Coombes 2016b (12mg,12mo)	185.6	46.4	33	50	8	28	5.0%	135.60 [119.49, 151.71]
Mashhadi 2018 (8mg,8w)	146	30	22	159	37	22	3.5%	-13.00 [-32.90, 6.90]
Nakagawa 2011 (12mg,12w)	196	15	10	204	43	10	1.8%	-8.00 [-36.23, 20.23]
Yoshida 2010b (12mg,12w)	225	29	15	212	25	15	3.6%	13.00 [-6.38, 32.38]
**Subtotal (95% CI)**			**113**			**103**	**15.9%**	**25.10 [-40.34, 90.54]**
Heterogeneity: Tau² = 5440.63; Chi² = 190.50, df = 4 (P < 0.00001); I² = 98%								
Test for overall effect: Z = 0.75 (P = 0.45)								
**3.5.3 13mg/day or more**								
Choi 2011 (20mg,12w)	169.8	3.19	14	178.3	28.8	13	5.2%	-8.50 [-24.24, 7.24]
Yoshida 2010c (18mg,12w)	233	35	16	212	25	15	3.1%	21.00 [-0.31, 42.31]
**Subtotal (95% CI)**			**30**			**28**	**8.3%**	**5.34 [-23.51, 34.19]**
Heterogeneity: Tau² = 343.75; Chi² = 4.76, df = 1 (P = 0.03); I² = 79%								
Test for overall effect: Z = 0.36 (P = 0.72)								
**Total (95% CI)**			**232**			**218**	**100.0%**	**7.98 [4.09, 11.88]**
Heterogeneity: Tau² = 11.08; Chi² = 284.00, df = 10 (P < 0.00001); I² = 96%								
Test for overall effect: Z = 4.02 (P < 0.0001)								
Test for subgroup differences: Chi² = 0.64, df = 2 (P = 0.73), I² = 0%								

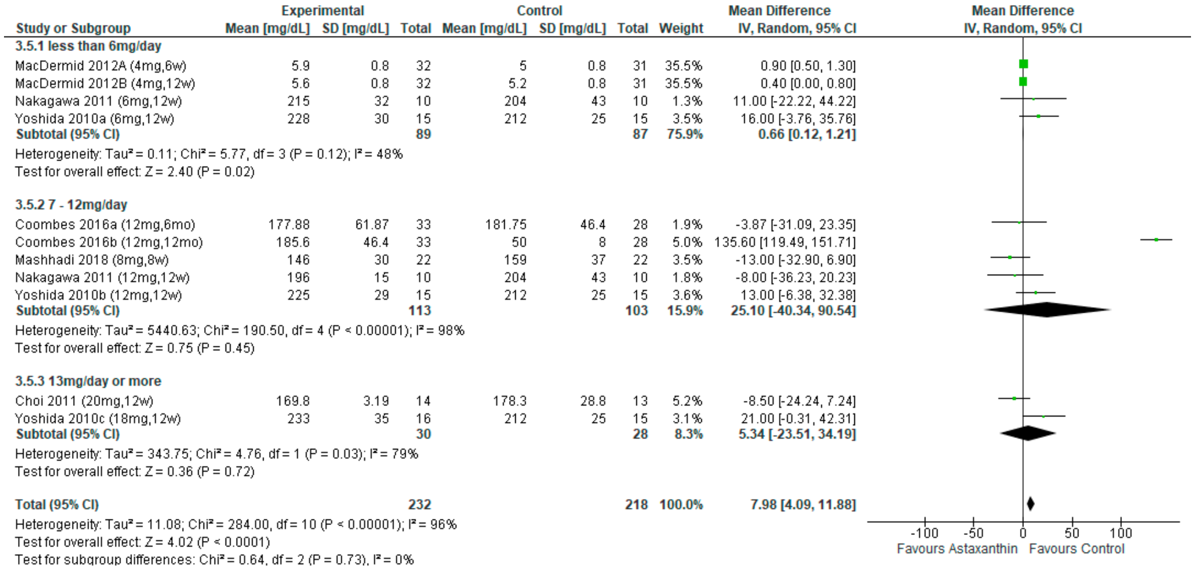

Figure S6b Subgroup meta-analysis of the effect of astaxanthin with different dosage on total cholesterol.

High-density lipoprotein cholesterol (HDL-C)

Study or Subgroup	Astaxanthin Mean [mg/dL]	Astaxanthin SD [mg/dL]	Astaxanthin Total	Control Mean [mg/dL]	Control SD [mg/dL]	Control Total	Weight	Mean Difference IV, Random, 95% CI
**2.6.1 8-week or less**								
MacDermid 2012A (4mg,6w)	1.2	0.3	32	1.2	0.4	31	50.5%	0.00 [-0.18, 0.18]
Mashhadi 2018 (8mg,8w)	38.1	5.7	22	36.1	7.2	22	0.6%	2.00 [-1.84, 5.84]
Sarkkinen 2018 (0.16mg,8w	57.23	19.34	18	66.51	16.63	17	0.1%	-9.28 [-21.21, 2.65]
**Subtotal (95% CI)**			**72**			**70**	**51.2%**	**0.14 [-2.38, 2.66]**
Heterogeneity: Tau² = 2.38; Chi² = 3.37, df = 2 (P = 0.19); I² = 41%								
Test for overall effect: Z = 0.11 (P = 0.91)								
**2.6.2 More than 8-week**								
Choi 2011 (20mg,12w)	50.4	12.6	14	50.2	8.44	13	0.1%	0.20 [-7.84, 8.24]
Coombes 2016a (12mg,6mo)	46.4	18.18	33	46.4	24.36	28	0.1%	0.00 [-10.95, 10.95]
Coombes 2016b (12mg,12mo)	50.27	17.79	33	44.47	18.56	28	0.1%	5.80 [-3.37, 14.97]
MacDermid 2012B (4mg,12w)	1.2	0.3	32	1.2	0.5	31	47.7%	0.00 [-0.20, 0.20]
Nakagawa 2011 (12mg,12w)	56.2	10.5	10	58.1	13	10	0.1%	-1.90 [-12.26, 8.46]
Nakagawa 2011 (6mg,12w)	65.5	14.7	10	58.1	13	10	0.1%	7.40 [-4.76, 19.56]
Yoshida 2010a (6mg,12w)	56	11	15	53	11	15	0.2%	3.00 [-4.87, 10.87]
Yoshida 2010b (12mg,12w)	63	8	15	53	11	15	0.2%	10.00 [3.12, 16.88]
Yoshida 2010c (18mg,12w)	55	8	16	53	11	15	0.2%	2.00 [-4.81, 8.81]
**Subtotal (95% CI)**			**178**			**165**	**48.8%**	**2.20 [-0.47, 4.87]**
Heterogeneity: Tau² = 4.79; Chi² = 12.06, df = 8 (P = 0.15); I² = 34%								
Test for overall effect: Z = 1.61 (P = 0.11)								
**Total (95% CI)**			**250**			**235**	**100.0%**	**0.05 [-0.26, 0.36]**
Heterogeneity: Tau² = 0.04; Chi² = 15.44, df = 11 (P = 0.16); I² = 29%								
Test for overall effect: Z = 0.29 (P = 0.77)								
Test for subgroup differences: Chi² = 1.21, df = 1 (P = 0.27), I² = 17.1%								

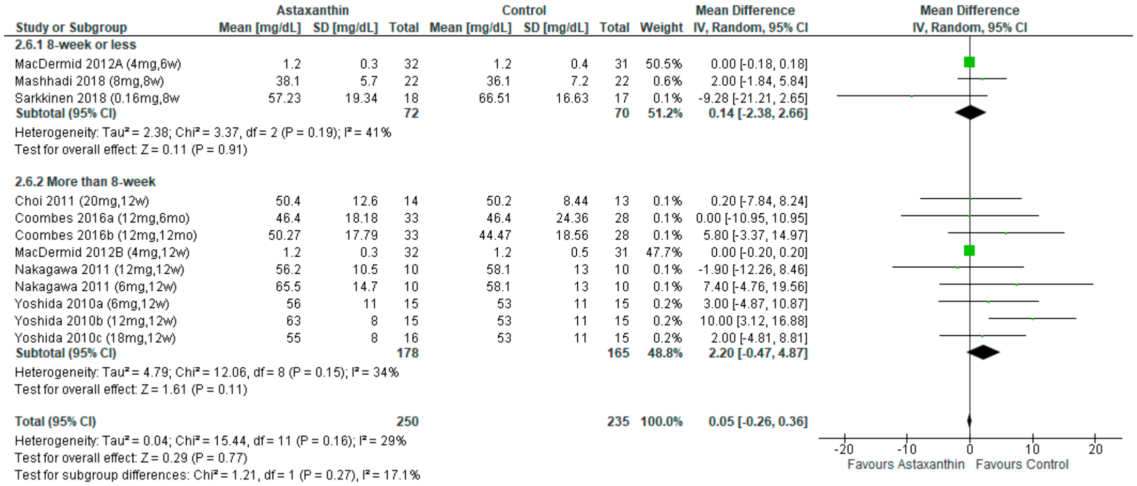

Figure S7a Subgroup meta-analysis of the effect of astaxanthin with different duration on high-density lipoprotein cholesterol.

Study or Subgroup	Astaxanthin Mean [mg/dL]	Astaxanthin SD [mg/dL]	Astaxanthin Total	Control Mean [mg/dL]	Control SD [mg/dL]	Control Total	Weight	Mean Difference IV, Random, 95% CI
**3.6.1 less than 6mg/day**								
MacDermid 2012A (4mg,6w)	1.2	0.3	32	1.2	0.4	31	50.5%	0.00 [-0.18, 0.18]
MacDermid 2012B (4mg,12w)	1.2	0.3	32	1.2	0.5	31	47.7%	0.00 [-0.20, 0.20]
Nakagawa 2011 (6mg,12w)	65.5	14.7	10	58.1	13	10	0.1%	7.40 [-4.76, 19.56]
Sarkkinen 2018 (0.16mg,8w	57.23	19.34	18	66.51	16.63	17	0.1%	-9.28 [-21.21, 2.65]
Yoshida 2010a (6mg,12w)	56	11	15	53	11	15	0.2%	3.00 [-4.87, 10.87]
**Subtotal (95% CI)**			**107**			**104**	**98.5%**	**0.00 [-0.15, 0.15]**
Heterogeneity: Tau² = 0.00; Chi² = 4.30, df = 4 (P = 0.37); I² = 7%								
Test for overall effect: Z = 0.01 (P = 0.99)								
**3.6.2 7 - 12mg/day**								
Coombes 2016a (12mg,6mo)	46.4	18.18	33	46.4	24.36	28	0.1%	0.00 [-10.95, 10.95]
Coombes 2016b (12mg,12mo)	50.27	17.79	33	44.47	18.56	28	0.1%	5.80 [-3.37, 14.97]
Mashhadi 2018 (8mg,8w)	38.1	5.7	22	36.1	7.2	22	0.6%	2.00 [-1.84, 5.84]
Nakagawa 2011 (12mg,12w)	56.2	10.5	10	58.1	13	10	0.1%	-1.90 [-12.26, 8.46]
Yoshida 2010b (12mg,12w)	63	8	15	53	11	15	0.2%	10.00 [3.12, 16.88]
**Subtotal (95% CI)**			**113**			**103**	**1.1%**	**3.63 [-0.30, 7.55]**
Heterogeneity: Tau² = 5.96; Chi² = 5.68, df = 4 (P = 0.22); I² = 30%								
Test for overall effect: Z = 1.81 (P = 0.07)								
**3.6.3 13mg/day or more**								
Choi 2011 (20mg,12w)	50.4	12.6	14	50.2	8.44	13	0.1%	0.20 [-7.84, 8.24]
Yoshida 2010c (18mg,12w)	55	8	16	53	11	15	0.2%	2.00 [-4.81, 8.81]
**Subtotal (95% CI)**			**30**			**28**	**0.4%**	**1.25 [-3.95, 6.44]**
Heterogeneity: Tau² = 0.00; Chi² = 0.11, df = 1 (P = 0.74); I² = 0%								
Test for overall effect: Z = 0.47 (P = 0.64)								
**Total (95% CI)**			**250**			**235**	**100.0%**	**0.05 [-0.26, 0.36]**
Heterogeneity: Tau² = 0.04; Chi² = 15.44, df = 11 (P = 0.16); I² = 29%								
Test for overall effect: Z = 0.29 (P = 0.77)								
Test for subgroup differences: Chi² = 3.49, df = 2 (P = 0.18), I² = 42.6%								

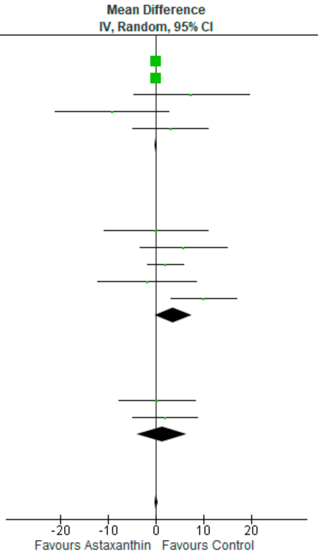

Figure S7b Subgroup meta-analysis of the effect of astaxanthin with different dosage on high-density lipoprotein cholesterol.

Low-density lipoprotein cholesterol (LDL-C)

Study or Subgroup	Astaxanthin Mean [mg/dL]	Astaxanthin SD [mg/dL]	Astaxanthin Total	Control Mean [mg/dL]	Control SD [mg/dL]	Control Total	Weight	Mean Difference IV, Random, 95% CI
**2.7.1 8-week or less**								
MacDermid 2012A (4mg,6w)	3.7	0.7	32	3	0.9	31	39.4%	0.70 [0.30, 1.10]
Mashhadi 2018 (8mg,8w)	88	27	22	92	26	22	0.0%	-4.00 [-19.66, 11.66]
Sarkkinen 2018 (0.16mg,8w	152.75	34.03	18	148.88	33.26	17	0.0%	3.87 [-18.43, 26.17]
**Subtotal (95% CI)**			**72**			**70**	**39.5%**	**0.70 [0.30, 1.10]**
Heterogeneity: Tau² = 0.00; Chi² = 0.42, df = 2 (P = 0.81); I² = 0%								
Test for overall effect: Z = 3.43 (P = 0.0006)								
**2.7.2 More than 8-week**								
Choi 2011 (20mg,12w)	114.6	28.6	14	114.8	24.1	13	0.0%	-0.20 [-20.10, 19.70]
Coombes 2016a (12mg,6mo)	96.67	52.2	33	108.28	51.43	28	0.0%	-11.61 [-37.69, 14.47]
Coombes 2016b (12mg,12mo)	104.4	46.4	33	102.47	53.36	28	0.0%	1.93 [-23.39, 27.25]
MacDermid 2012B (4mg,12w)	3.6	0.6	32	3	0.7	31	60.4%	0.60 [0.28, 0.92]
Nakagawa 2011 (12mg,12w)	112	19	10	122	37	10	0.0%	-10.00 [-35.78, 15.78]
Nakagawa 2011 (6mg,12w)	124	29	10	122	37	10	0.0%	2.00 [-27.14, 31.14]
Yoshida 2010a (6mg,12w)	150	28	15	138	32	15	0.0%	12.00 [-9.52, 33.52]
Yoshida 2010b (12mg,12w)	144	32	15	138	32	15	0.0%	6.00 [-16.90, 28.90]
Yoshida 2010c (18mg,12w)	159	31	16	138	32	15	0.0%	21.00 [-1.20, 43.20]
**Subtotal (95% CI)**			**178**			**165**	**60.5%**	**0.60 [0.28, 0.93]**
Heterogeneity: Tau² = 0.00; Chi² = 6.05, df = 8 (P = 0.64); I² = 0%								
Test for overall effect: Z = 3.68 (P = 0.0002)								
**Total (95% CI)**			**250**			**235**	**100.0%**	**0.64 [0.39, 0.89]**
Heterogeneity: Tau² = 0.00; Chi² = 6.60, df = 11 (P = 0.83); I² = 0%								
Test for overall effect: Z = 5.02 (P < 0.00001)								
Test for subgroup differences: Chi² = 0.13, df = 1 (P = 0.72), I² = 0%								

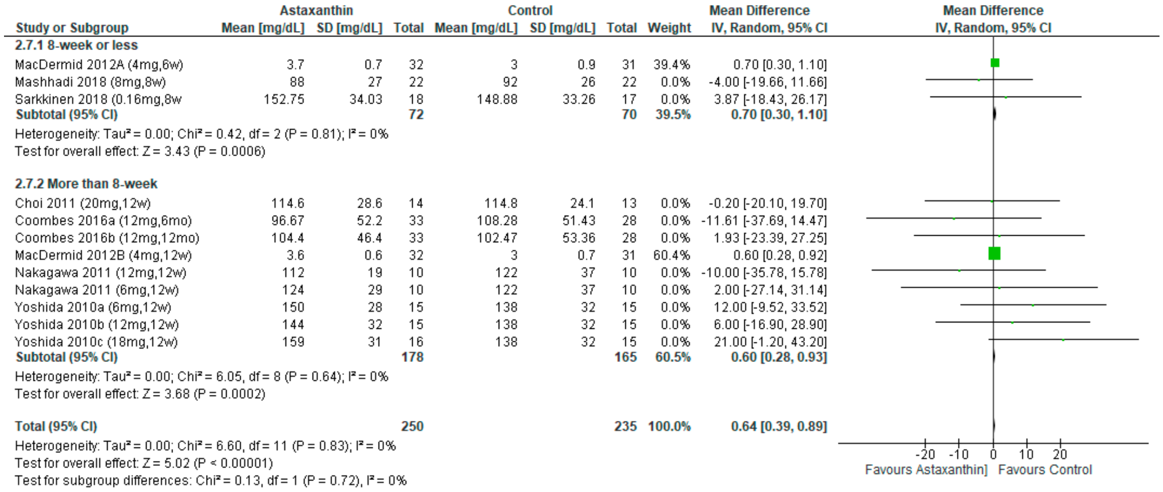

Figure S8a Subgroup meta-analysis of the effect of astaxanthin with different duration on low-density lipoprotein cholesterol.

Study or Subgroup	Astaxanthin Mean [mg/dL]	Astaxanthin SD [mg/dL]	Astaxanthin Total	Control Mean [mg/dL]	Control SD [mg/dL]	Control Total	Weight	Mean Difference IV, Random, 95% CI
**3.7.1 less than 6mg/day**								
MacDermid 2012A (4mg,6w)	3.7	0.7	32	3	0.9	31	39.4%	0.70 [0.30, 1.10]
MacDermid 2012B (4mg,12w)	3.6	0.6	32	3	0.7	31	60.4%	0.60 [0.28, 0.92]
Nakagawa 2011 (6mg,12w)	124	29	10	122	37	10	0.0%	2.00 [-27.14, 31.14]
Sarkkinen 2018 (0.16mg,8w	152.75	34.03	18	148.88	33.26	17	0.0%	3.87 [-18.43, 26.17]
Yoshida 2010a (6mg,12w)	150	28	15	138	32	15	0.0%	12.00 [-9.52, 33.52]
**Subtotal (95% CI)**			**107**			**104**	**99.9%**	**0.64 [0.39, 0.89]**
Heterogeneity: Tau² = 0.00; Chi² = 1.31, df = 4 (P = 0.86); I² = 0%								
Test for overall effect: Z = 5.02 (P < 0.00001)								
**3.7.2 7 - 12mg/day**								
Coombes 2016a (12mg,6mo)	96.67	52.2	33	108.28	51.43	28	0.0%	-11.61 [-37.69, 14.47]
Coombes 2016b (12mg,12mo)	104.4	46.4	33	102.47	53.36	28	0.0%	1.93 [-23.39, 27.25]
Mashhadi 2018 (8mg,8w)	88	27	22	92	26	22	0.0%	-4.00 [-19.66, 11.66]
Nakagawa 2011 (12mg,12w)	112	19	10	122	37	10	0.0%	-10.00 [-35.78, 15.78]
Yoshida 2010b (12mg,12w)	144	32	15	138	32	15	0.0%	6.00 [-16.90, 28.90]
**Subtotal (95% CI)**			**113**			**103**	**0.1%**	**-3.23 [-12.98, 6.52]**
Heterogeneity: Tau² = 0.00; Chi² = 1.45, df = 4 (P = 0.83); I² = 0%								
Test for overall effect: Z = 0.65 (P = 0.52)								
**3.7.3 13mg/day or more**								
Choi 2011 (20mg,12w)	114.6	28.6	14	114.8	24.1	13	0.0%	-0.20 [-20.10, 19.70]
Yoshida 2010c (18mg,12w)	159	31	16	138	32	15	0.0%	21.00 [-1.20, 43.20]
**Subtotal (95% CI)**			**30**			**28**	**0.0%**	**9.81 [-10.94, 30.55]**
Heterogeneity: Tau² = 109.00; Chi² = 1.94, df = 1 (P = 0.16); I² = 49%								
Test for overall effect: Z = 0.93 (P = 0.35)								
**Total (95% CI)**			**250**			**235**	**100.0%**	**0.64 [0.39, 0.89]**
Heterogeneity: Tau² = 0.00; Chi² = 6.60, df = 11 (P = 0.83); I² = 0%								
Test for overall effect: Z = 5.02 (P < 0.00001)								
Test for subgroup differences: Chi² = 1.36, df = 2 (P = 0.51), I² = 0%								

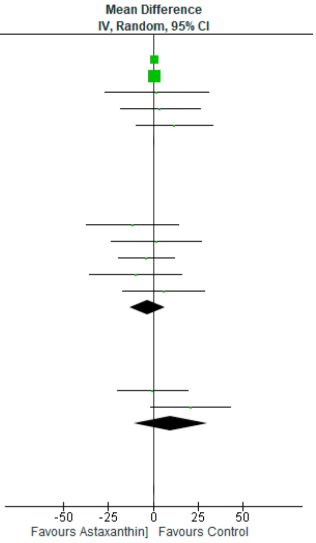

Figure S8b Subgroup meta-analysis of the effect of astaxanthin with different dosage on low-density lipoprotein cholesterol.

Triglyceride (TG)

Study or Subgroup	Astaxanthin Mean [mg/dL]	Astaxanthin SD [mg/dL]	Astaxanthin Total	Control Mean [mg/dL]	Control SD [mg/dL]	Control Total	Weight	Mean Difference IV, Random, 95% CI
**2.8.1 8-week or less**								
MacDermid 2012A (4mg,6w)	2.1	0.8	32	1.8	1.1	31	51.9%	0.30 [-0.18, 0.78]
Mashhadi 2018 (8mg,8w)	128	52	22	150	85	22	0.1%	-22.00 [-63.64, 19.64]
Sarkkinen 2018 (0.16mg,8w	128.43	63.77	18	109.83	38.97	17	0.2%	18.60 [-16.20, 53.40]
**Subtotal (95% CI)**			**72**			**70**	**52.2%**	**0.41 [-6.97, 7.79]**
Heterogeneity: Tau² = 15.24; Chi² = 2.16, df = 2 (P = 0.34); I² = 8%								
Test for overall effect: Z = 0.11 (P = 0.91)								
**2.8.2 More than 8-week**								
Choi 2011 (20mg,12w)	110.9	38.4	14	119.2	65.9	13	0.1%	-8.30 [-49.38, 32.78]
Coombes 2016a (12mg,6mo)	124	132.86	33	159.43	168.3	28	0.0%	-35.43 [-112.51, 41.65]
Coombes 2016b (12mg,12mo)	132.86	70.86	33	177.15	150.58	28	0.1%	-44.29 [-105.08, 16.50]
MacDermid 2012B (4mg,12w)	1.9	1.1	32	2.5	2	31	46.9%	-0.60 [-1.40, 0.20]
Yoshida 2010a (6mg,12w)	125	41	15	140	40	15	0.2%	-15.00 [-43.99, 13.99]
Yoshida 2010b (12mg,12w)	110	44	15	140	40	15	0.2%	-30.00 [-60.09, 0.09]
Yoshida 2010c (18mg,12w)	112	40	16	140	40	15	0.3%	-28.00 [-56.18, 0.18]
**Subtotal (95% CI)**			**158**			**145**	**47.8%**	**-15.25 [-29.75, -0.75]**
Heterogeneity: Tau² = 145.69; Chi² = 11.12, df = 6 (P = 0.08); I² = 46%								
Test for overall effect: Z = 2.06 (P = 0.04)								
**Total (95% CI)**			**230**			**215**	**100.0%**	**-0.34 [-1.76, 1.08]**
Heterogeneity: Tau² = 0.95; Chi² = 17.45, df = 9 (P = 0.04); I² = 48%								
Test for overall effect: Z = 0.47 (P = 0.64)								
Test for subgroup differences: Chi² = 3.56, df = 1 (P = 0.06), I² = 71.9%								

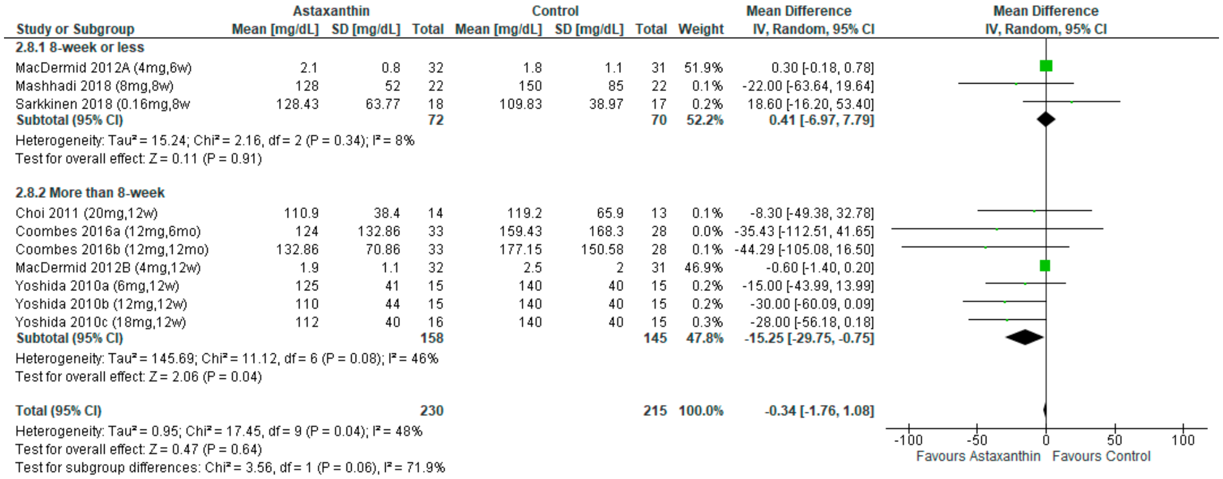

Figure S9a Subgroup meta-analysis of the effect of astaxanthin with different duration on triglyceride.

Study or Subgroup	Astaxanthin Mean [mg/dL]	Astaxanthin SD [mg/dL]	Astaxanthin Total	Control Mean [mg/dL]	Control SD [mg/dL]	Control Total	Weight	Mean Difference IV, Random, 95% CI
**3.8.1 less than 6mg/day**								
Sarkkinen 2018 (0.16mg,8w	128.43	63.77	18	109.83	38.97	17	0.2%	18.60 [-16.20, 53.40]
Yoshida 2010a (6mg,12w)	125	41	15	140	40	15	0.2%	-15.00 [-43.99, 13.99]
MacDermid 2012B (4mg,12w)	1.9	1.1	32	2.5	2	31	46.9%	-0.60 [-1.40, 0.20]
MacDermid 2012A (4mg,6w)	2.1	0.8	32	1.8	1.1	31	51.9%	0.30 [-0.18, 0.78]
**Subtotal (95% CI)**			**97**			**94**	**99.2%**	**-0.09 [-0.98, 0.79]**
Heterogeneity: Tau² = 0.31; Chi² = 5.71, df = 3 (P = 0.13); I² = 47%								
Test for overall effect: Z = 0.21 (P = 0.84)								
**3.8.2 7 - 12mg/day**								
Coombes 2016a (12mg,6mo)	124	132.86	33	159.43	168.3	28	0.0%	-35.43 [-112.51, 41.65]
Coombes 2016b (12mg,12mo)	132.86	70.86	33	177.15	150.58	28	0.1%	-44.29 [-105.08, 16.50]
Mashhadi 2018 (8mg,8w)	128	52	22	150	85	22	0.1%	-22.00 [-63.64, 19.64]
Yoshida 2010b (12mg,12w)	110	44	15	140	40	15	0.2%	-30.00 [-60.09, 0.09]
**Subtotal (95% CI)**			**103**			**93**	**0.4%**	**-30.08 [-51.80, -8.36]**
Heterogeneity: Tau² = 0.00; Chi² = 0.37, df = 3 (P = 0.95); I² = 0%								
Test for overall effect: Z = 2.71 (P = 0.007)								
**3.8.3 13mg/day or more**								
Choi 2011 (20mg,12w)	110.9	38.4	14	119.2	65.9	13	0.1%	-8.30 [-49.38, 32.78]
Yoshida 2010c (18mg,12w)	112	40	16	140	40	15	0.3%	-28.00 [-56.18, 0.18]
**Subtotal (95% CI)**			**30**			**28**	**0.4%**	**-21.70 [-44.93, 1.54]**
Heterogeneity: Tau² = 0.00; Chi² = 0.60, df = 1 (P = 0.44); I² = 0%								
Test for overall effect: Z = 1.83 (P = 0.07)								
**Total (95% CI)**			**230**			**215**	**100.0%**	**-0.34 [-1.76, 1.08]**
Heterogeneity: Tau² = 0.95; Chi² = 17.45, df = 9 (P = 0.04); I² = 48%								
Test for overall effect: Z = 0.47 (P = 0.64)								
Test for subgroup differences: Chi² = 10.61, df = 2 (P = 0.005), I² = 81.2%								

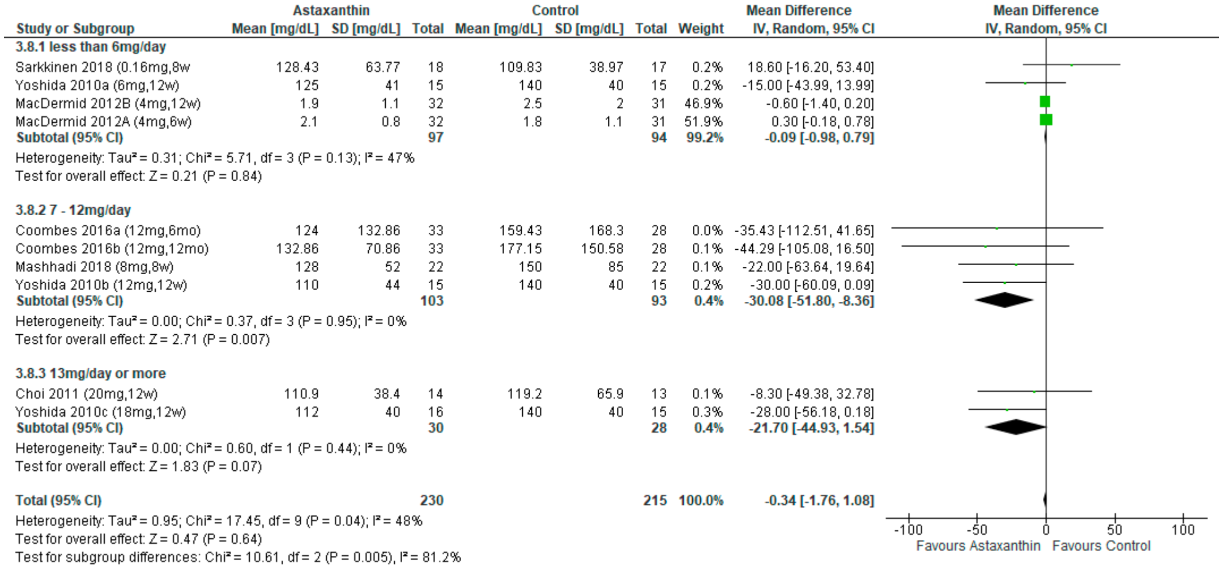

Figure S9b Subgroup meta-analysis of the effect of astaxanthin with different dosage on triglyceride.
